# Long-term preservation of biomolecules in lake sediments: potential importance of physical shielding by recalcitrant cell walls

**DOI:** 10.1093/pnasnexus/pgac076

**Published:** 2022-06-08

**Authors:** Xingguo Han, Julie Tolu, Longhui Deng, Annika Fiskal, Carsten Johnny Schubert, Lenny H E Winkel, Mark Alexander Lever

**Affiliations:** Institute of Biogeochemistry and Pollutant Dynamics, Swiss Federal Institute of Technology, Zurich (ETH Zurich), Universitätstrasse 16, 8092 Zurich, Switzerland; Institute of Biogeochemistry and Pollutant Dynamics, Swiss Federal Institute of Technology, Zurich (ETH Zurich), Universitätstrasse 16, 8092 Zurich, Switzerland; Department of Water Resources and Drinking Water, Swiss Federal Institute of Aquatic Science and Technology (EAWAG), Überlandstrasse 133, 8600 Dübendorf, Switzerland; Institute of Biogeochemistry and Pollutant Dynamics, Swiss Federal Institute of Technology, Zurich (ETH Zurich), Universitätstrasse 16, 8092 Zurich, Switzerland; Institute of Biogeochemistry and Pollutant Dynamics, Swiss Federal Institute of Technology, Zurich (ETH Zurich), Universitätstrasse 16, 8092 Zurich, Switzerland; Institute of Biogeochemistry and Pollutant Dynamics, Swiss Federal Institute of Technology, Zurich (ETH Zurich), Universitätstrasse 16, 8092 Zurich, Switzerland; Department of Surface Waters - Research and Management, Swiss Federal Institute of Aquatic Science and Technology (EAWAG), Seestrasse 79, 6047 Kastanienbaum, Switzerland; Institute of Biogeochemistry and Pollutant Dynamics, Swiss Federal Institute of Technology, Zurich (ETH Zurich), Universitätstrasse 16, 8092 Zurich, Switzerland; Department of Water Resources and Drinking Water, Swiss Federal Institute of Aquatic Science and Technology (EAWAG), Überlandstrasse 133, 8600 Dübendorf, Switzerland; Institute of Biogeochemistry and Pollutant Dynamics, Swiss Federal Institute of Technology, Zurich (ETH Zurich), Universitätstrasse 16, 8092 Zurich, Switzerland

**Keywords:** lake sediments, biomolecules, DNA, long-term preservation, physical shielding

## Abstract

Even though lake sediments are globally important organic carbon (OC) sinks, the controls on long-term OC storage in these sediments are unclear. Using a multiproxy approach, we investigate changes in diatom, green algae, and vascular plant biomolecules in sedimentary records from the past centuries across five temperate lakes with different trophic histories. Despite past increases in the input and burial of OC in sediments of eutrophic lakes, biomolecule quantities in sediments of all lakes are primarily controlled by postburial microbial degradation over the time scales studied. We, moreover, observe major differences in biomolecule degradation patterns across diatoms, green algae, and vascular plants. Degradation rates of labile diatom DNA exceed those of chemically more resistant diatom lipids, suggesting that chemical reactivity mainly controls diatom biomolecule degradation rates in the lakes studied. By contrast, degradation rates of green algal and vascular plant DNA are significantly lower than those of diatom DNA, and in a similar range as corresponding, much less reactive lipid biomarkers and structural macromolecules, including lignin. We propose that physical shielding by degradation-resistant cell wall components, such as algaenan in green algae and lignin in vascular plants, contributes to the long-term preservation of labile biomolecules in both groups and significantly influences the long-term burial of OC in lake sediments.

Significance Statement Lake sediments are globally important OC sinks, yet the factors controlling the contributions of different organisms and their biomass components to sedimentary OC burial are not well-understood. We show that diatom, green algal, and vascular plant biomolecules follow different trajectories over time scales of centuries in lake sediments. While the degradation of diatom biomass follows the chemical reactivities of its biomolecules, the degradation of green algal and vascular plant biomolecules is much slower and does not vary in relation to chemical reactivity. We propose that degradation-resistant cell walls in certain green algae and vascular plants effectively protect biomass of these organisms from degradation and contribute significantly to the long-term burial of OC in lake sediments.

## Introduction

Even though lakes and reservoirs only account for 2% of the Earth's surface, the global annual burial of organic carbon (OC) in lake and reservoir sediments (0.15 Pg C year^–1^) is comparable to that in ocean sediments (0.2 Pg C year^–1^) (1, 2). A major fraction of the OC that enters lake sediments is microbially respired to the greenhouse gas methane. This methane from lake sediments contributes significantly to the ∼32% of annual natural and ∼17% of annual global methane emissions to the atmosphere that are released by freshwater sediments (3). Despite this worldwide importance of lake sediments as OC sinks and greenhouse gas sources, the factors controlling whether lake sedimentary OC remains buried or is microbially converted to methane are not well understood (1, 4).

Over the past century, the anthropogenic release of nutrients, such as nitrogen (N) and phosphorus (P), from sewage, detergents, or agriculture has widely enhanced lake water column primary production and OC loading and led to eutrophication (4–6). Resulting increases in turbidity and decreases in dissolved oxygen (O_2_) concentrations have altered lake ecosystems and negatively impacted fisheries (7), while increasing OC deposition and even OC burial in lake sediments (4, 5). In addition, land-use changes, e.g. deforestation or dam construction, have impacted lakes by altering inputs of land-derived OC (8).

Sedimentary biomarker records are frequently used to study past environmental changes in lakes and lake catchments (9–11). Saturated short-chain *n*-alkanes (C_15_+C_17_+C_19_) and fatty acids (C_14_+C_16_+C_18_) provide insights into past microalgal primary production (12) and contributions of microalgal groups. For instance, diatoms have a characteristically higher content of the polyunsaturated fatty acid C_20:5n-3_ than C_22:6n-3_ (10). Similarly, the sterols brassicasterol and 24-methylenecholesterol originate primarily from diatoms (13, 14). Green algae (*Chlorophyta*) have elevated contents of the C_18_ polyunsaturated fatty acids C_18:2n-6_ and C_18:3n-3_ (10). Vascular plant-derived saturated long-chain *n*-alkanes (C_27_+C_29_+C_31_) and fatty acids (C_24_+C_26_+C_28_), and lignin phenols serve as proxies for terrestrial plant inputs and land use changes (15, 16). Challenges in the interpretation of biomarker records arise from the fact that biomarkers differ in chemical reactivity (17), and that many biomarkers are not unique to one organism group (10). Moreover, insights into environmental conditions at the time of sediment deposition can be biased by input of fossil biomarkers, e.g. from the erosion of much older soil sequences (18, 19).

Sedimentary macromolecule structures, analyzed by pyrolysis gas chromatography–mass spectrometry (Py–GC/MS), are also used to study past changes in lake ecosystems (20, 21). By thermally breaking or volatilizing organic macromolecules, dominant compound classes, e.g. carbohydrates, proteins, and lipids, can be identified in a single measurement (21, 22). This, combined with the ability to extract and analyze compounds that are resistant to extraction by chemical hydrolysis methods (23), makes Py–GC/MS a powerful and high-throughput tool to characterize organic macromolecules in complex soil or sedimentary matrices. Limitations include lower source specificity than biomarkers, and that the same products can be generated from different compounds, e.g. pyrrole can be released during the pyrolysis of both proteins and chlorophyll (24).

In addition to biomarkers and Py–GC/MS, sedimentary DNA has in recent years increasingly been used to study past environmental changes (25, 26). DNA sequences have the distinct advantage over the other analyses that they can reveal the precise phylogenetic identities of the source organisms (27). Genetic records of terrestrial plants and certain algae (e.g. diatoms, dinoflagellates, and green algae) can be preserved for 10,000 years in lake sediments (28–30). By contrast, DNA of most Bacteria and Archaea and certain eukaryotic phytoplankton groups is more rapidly mineralized after death (31, 32).

The factors that determine biomolecule degradation in sediments are diverse and vary as a function of time (33–35). Adsorption by electrostatic interactions with mineral surfaces (36, 37) and complexation or aggregation with other organic compounds, e.g. humic substances or proteins, slows DNA degradation by restricting enzymatic access (38), though neither may support the long-term preservation of DNA polymers (31). Inherent variations in chemical reactivities, e.g. due to structural differences between membrane lipids, can also in some cases explain variations in biomolecule compositions through time (17, 39). Biotic exclusion, whereby microbial or enzymatic access to biomolecules is effectively blocked, may also influence biomolecule degradation and even preserve labile organic compounds over geologic time scales (40, 41). Mineral protection, e.g. within enzymatically inaccessible pore space or via covalent formation or chelation to solid-phase mineral elements, are potential key biotic exclusion mechanisms (35, 41). In addition, shielding (encapsulation) by enzymatically resistant cell wall structures may result in long-term preservation of labile organic compounds long after death in certain organisms (31, 42). However, little is known about how OC preservation mechanisms compare across different organisms and biomolecule groups, and how these differences in preservation mechanisms influence the long-term contributions of different organisms to sedimentary OC sinks. To address these questions, comparisons of biomolecule inventories within and between organism groups through time provide useful insights.

**Fig. 1.**
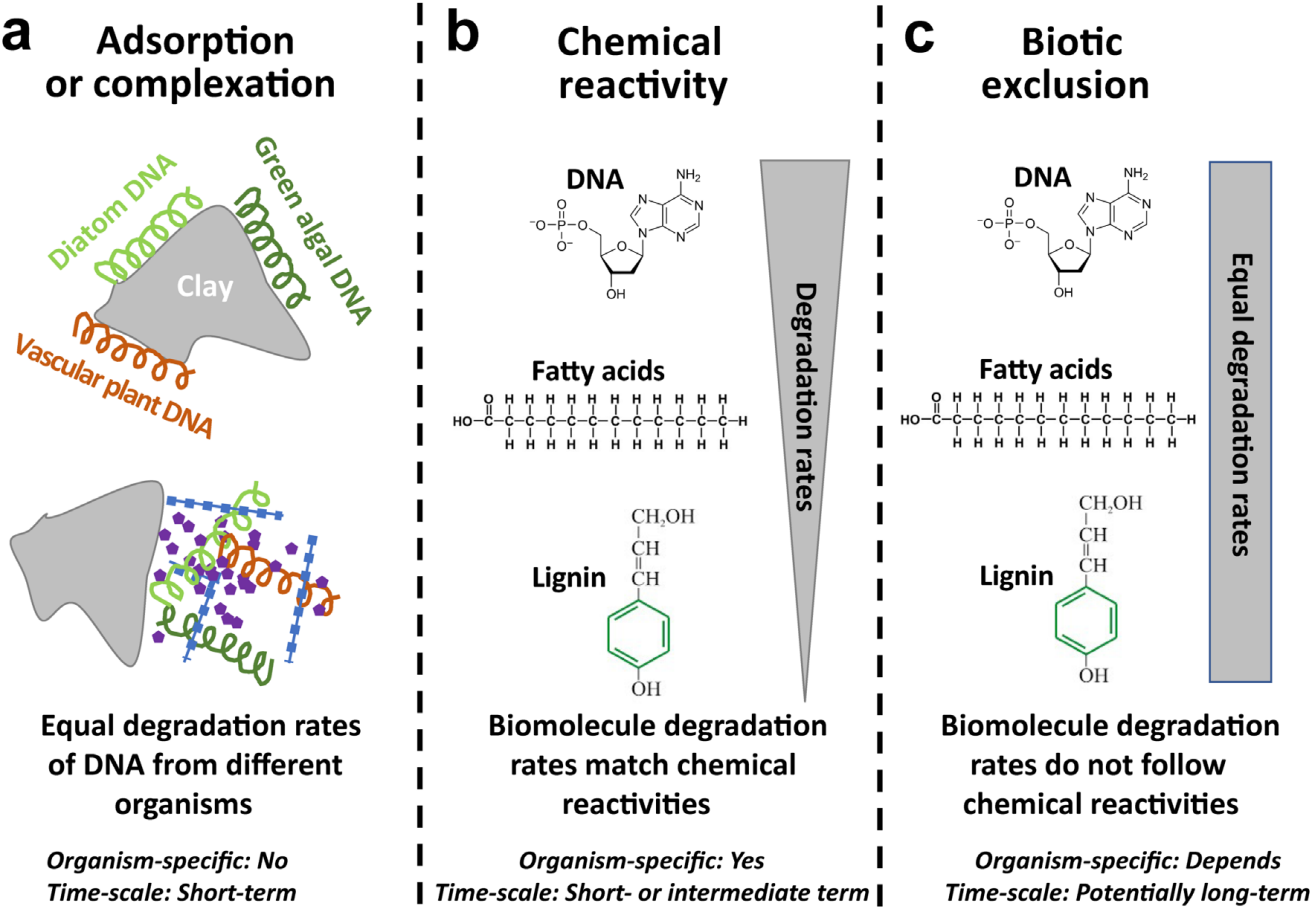
Conceptual sketch showing three scenarios to explain biomolecule degradation trends in lake sediments through time. (a) Electrostatic adsorption to minerals (top) or complexation (aggregation) with minerals or organic compounds (bottom) control DNA degradation rates. (b) Chemical reactivities at the compound-level determine degradation rates, and vary between organisms based on their chemical building blocks. (c) Biotic exclusion causes chemically reactive (labile) compounds to have similar degradation rates as chemically resistant compounds. Degradation rates can vary between organisms, e.g. if biotic exclusion is driven by chemically resistant cell walls which differ between organisms, or if mineral protection was initiated in physicochemically different habitats, such as soils vs. sediments.

Here, we use a multiproxy approach targeting DNA, biomarkers, and OC macromolecular structures of three dominant phototrophic organism groups to explore the controls on organic biomolecule degradation in lake sediments. We study sedimentary records of five lakes in central Switzerland that have well-established trophic histories (for details see “Background and environmental context” in Materials and Methods), including past increases in sedimentary OC burial in response to eutrophication (4). The five lakes include the currently oligotrophic Lake Lucerne, the mesotrophic Lake Zurich, and the eutrophic Lake Greifen, Lake Baldegg, and Lake Zug. Based on DNA, lipid biomarker, and organic macromolecule records from the past centuries, we investigate the impact of historic changes in trophic state on sedimentary biomolecule inventories of diatoms (*Bacillariophyta*), green algae (*Chlorophyta*), and vascular plants (*Tracheophyta*). These organism groups share labile intracellular biomolecules (e.g. DNA) but differ in compositions of chemically less reactive cell membrane and cell wall molecules (e.g. lipids, algaenan, and lignin). In addition, we explore first-order relationships between organic matter content and sediment age (43) to estimate degradation rates and degradation controls of diatom, green algal, and vascular plant DNA, lipids and macromolecules through time. We hypothesize that DNA protection by electrostatic adsorption and/or complexation results in similar degradation rates of DNA across different organisms due to the universal chemical structure of DNA (Fig. 1a). We, moreover, postulate that, if differences in chemical reactivity drive biomolecule degradation rates, then biomolecule inventories through time should reflect these differences (Fig. 1b). Finally, if biotic exclusion (physical shielding) is a key driver of biomolecule degradation, then degradation rates of physically protected labile biomolecules, such as DNA, should resemble those of more degradation-resistant biomass components (Fig. 1c).

We examine diatom, green algae, and vascular plant biomolecule contents through time based on quantitative polymerase chain reaction (qPCR) and next-generation sequencing of chloroplast genes encoding the large subunit of ribulose-1,5-bisphosphate carboxylase (*rbc*L), and nuclear genes encoding eukaryotic 18S rRNA. We then compare DNA data to inventories and degradation rates of biomarkers (short- and long-chain fatty acids and *n-*alkanes, algal group-specific fatty acids and sterols, and lignin), as well as organic macromolecules determined by Py–GC/MS. Our multiproxy approach produces novel insights into the source-specific controls and time scales over which OC is degraded in lake sediments.

## Results

### Postdepositional trends in biomolecule inventories across trophic states

**Fig. 2.**
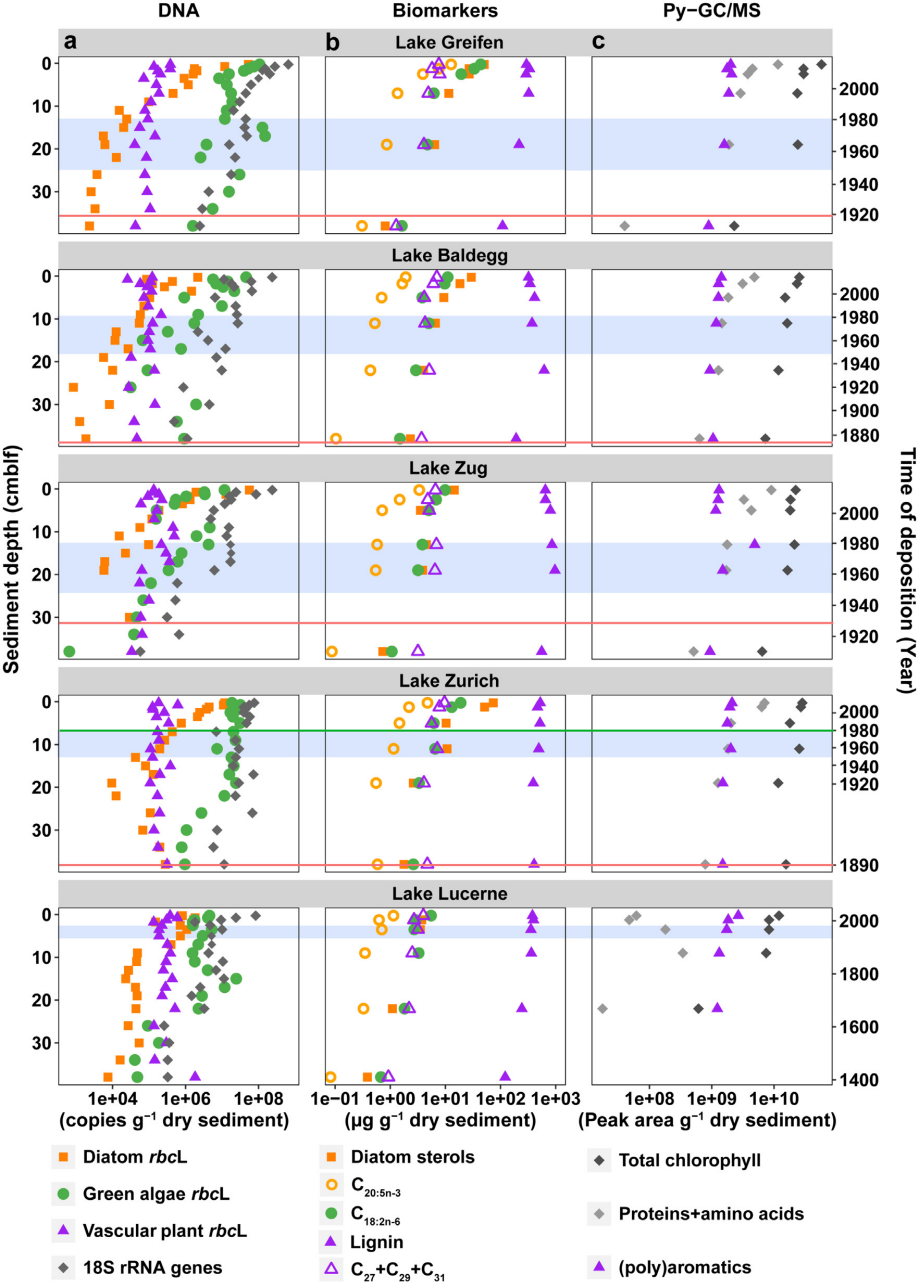
Sediment depth and age profiles of (a) group-specific *rbc*L copy numbers, and total 18S rRNA gene copy numbers, (b) lipid biomarker and lignin contents, (c) organic macromolecule contents determined by Py–GC/MS, all for the deepest station in each of the five lakes. Lakes are listed in order of present-day trophic state, from most eutrophic (Lake Greifen) to oligotrophic (Lake Lucerne). The onset of eutrophication is indicated by the red lines (Lake Greifen: ∼1920; Lake Baldegg: ∼1890; Lake Zug: ∼1930; and Lake Zurich: ∼1890). The time period of peak eutrophication from 1950 to 1980 is indicated as a blue-shaded area. The transition from eutrophic to mesotrophic in Lake Zurich took place around 1980 (green line; note: the increased sedimentation rates in Lake Zurich below 19 cm (∼1920), which are the result of several large turbidites.). Sediment age models of all sites based on extrapolated radionuclide (Pb-210_unsupported_ and Cs-137) measurements were previously published (4). *rbc*L and 18S rRNA gene copy numbers were also produced for the shallow and medium water depth stations in each lake (Figures S2 and S3, Supplementary Material).

Depth- and age-related trends in *rbc*L copy numbers of diatoms, green algae, and vascular plants, and total eukaryotic 18S rRNA gene copy numbers differ from each other but are remarkably similar, i.e. strongly overlapping, within and across the five lakes, despite strong differences in lake trophic histories (deep station: Fig. 2a; all three stations: Figures S2 and S3, Supplementary Material). Matching their present-day conditions, eu- and mesotrophic lakes have the highest diatom and green algal *rbc*L copy numbers at the sediment surface. Yet, this trend does not hold downcore, e.g. deep layers of Lakes Greifen and Baldegg that were deposited under already eutrophic conditions have the lowest diatom *rbc*L copy numbers of all lakes.

Diatom *rbc*L copy numbers decrease strongly with depth, from, on average, ∼10^8^ at the top to ∼10^4^ g^–1^ at the bottom of cores, with the steepest decrease (∼10^3^-fold) in the top 10 cm. Copy numbers of green algal *rbc*L, which are more scattered than those of diatoms, also decrease with depth and age, though more gradually, and thus generally exceed those of diatoms below 5 cm. Subsurface peaks in green algal, but not diatom, *rbc*L copy numbers are present in sediment layers deposited around or shortly after the period of peak eutrophication in Lake Greifen and Lake Zug, and in a layer of Lake Lucerne from ∼16 cm that was deposited during the latter half of the 18th century. *rbc*L copy numbers of green algae are, moreover, stable in the top ∼20 cm of Lake Zurich, corresponding approximately to the time since which bottom water at this station has been hypoxic. By comparison, *rbc*L copy numbers of vascular plants vary less between lakes and are remarkably stable throughout all sediment cores (∼10^5^ to ∼10^6^ g^–1^ dry sediment), even going back ∼600 years in Lake Lucerne. Gene copy numbers of 18S rRNA genes generally exceed those of *rbc*L and show an intermediate trend compared to the three *rbc*L targets, declining by ∼2 orders in the top 5 cm, and by ∼1 order of magnitude in the remainder of cores (from ∼10^9^ to ∼10^6^ copies g^–1^ dry sediment). Local subsurface peaks in 18S rRNA gene copy numbers were observed in the same layers as for green algae. Also, as was observed for green algal *rcb*L copy numbers, 18S rRNA gene copy numbers have remained stable at the deep station in Lake Zurich since the early 20th century.

Similar to microalgal *rbc*L copy numbers, contents of the mainly diatom-derived C_20:5n-3_ fatty acid and ‘diatom sterols’, and of the green algal biomarker C_18:2n-6_ fatty acid decrease with sediment depth and time (Fig. 2b). This is also the case for total chlorophyll and proteins (Fig. 2c), identified from the pyrolysis products “phytadienes+phytenes+pristenes” (chlorophyll and its degradation products) and “diketopiperazines” (proteins plus nonprotein amino acids), which mainly derive from microalgae (44, 45). By contrast, as with vascular plant *rbc*L, the contents of mainly terrestrial plant-derived long chain *n*-alkanes (C_27_+C_29_+C_31_), lignin, and (poly)aromatics (dihydro-indenone and 9-methylene-fluorene) are stable with sediment depth and age (Fig. 2b and c). Other, less group-specific biomarkers, such as the mainly microalgae-derived chlorophyll *a*, short-chain *n*-alkanes (C_15_+C_17_+C_19_) and short-chain fatty acids (C_14_+C_16_+C_18_), and the mainly vascular plant-derived long chain fatty acids (C_24_+C_26_+C_28_; Figure S4, Supplementary Material), show trends comparable to microalgal and vascular plant biomarkers in Fig. 2.

As with different DNA targets, biomarkers and organic macromolecules differ greatly between each other in vertical profiles. Yet, within each biomarker or macromolecule group vertical profiles between lakes are similar, independent of trophic state. Hereby, microalgae-derived compounds show their biggest changes (decreases) with sediment age, consistent with known first-order relationships between OC content and sediment age (43, 46). In the following, we explore sediment age as a unifying variable that enables general inferences regarding the degradation rates and degradation controls of chemically diverse biomolecules across lakes that differ in trophic state.

### Degradation rates of DNA, biomarkers, and macromolecules through time

**Fig. 3.**
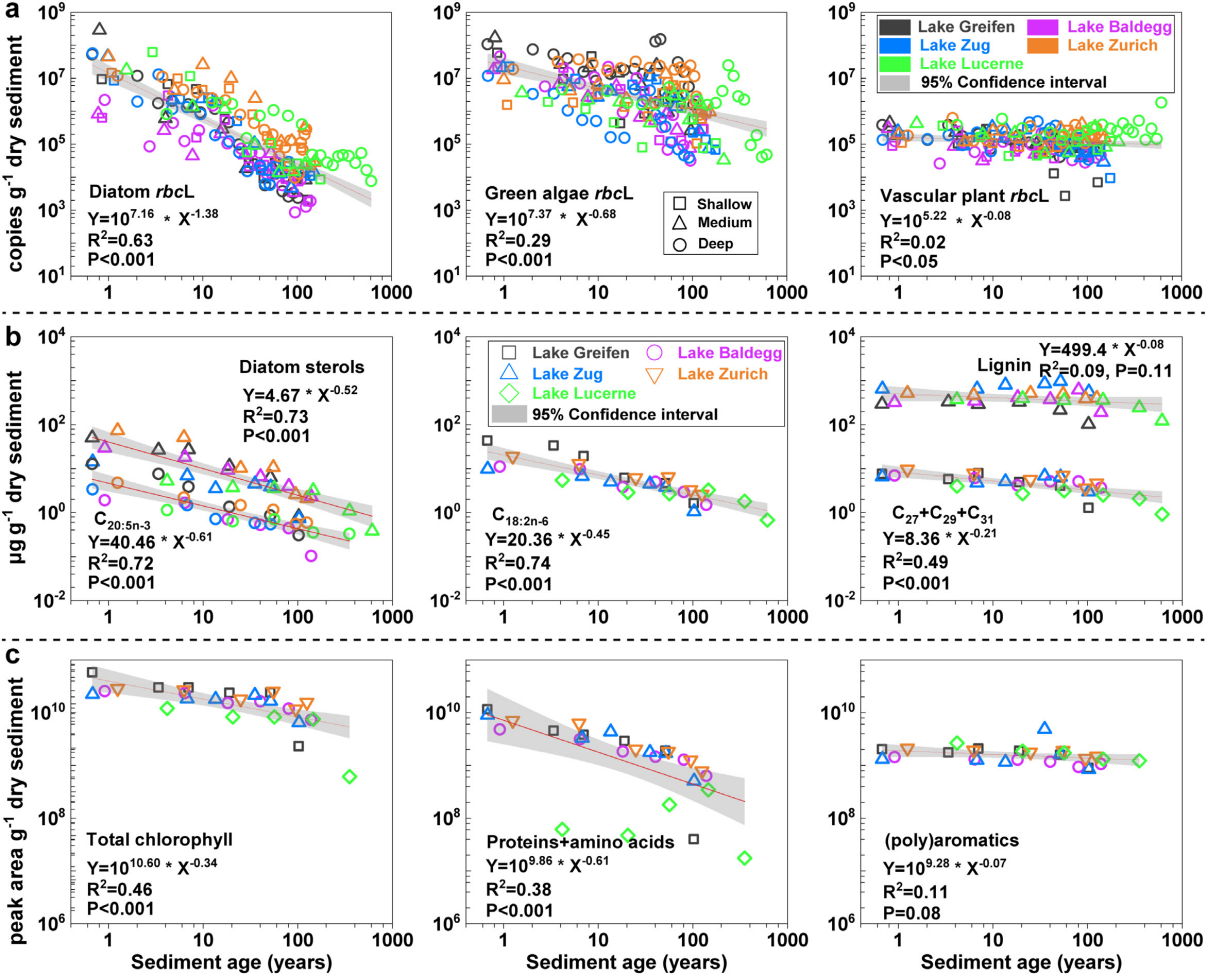
Relationships between sediment age and (a) *rbc*L copy numbers of diatoms, green algae, and vascular plants, (b) biomarker contents, and (c) organic macromolecule contributions determined by Py–GC/MS (all parameters normalized to g dry sediment). Best fit lines are based on power functions. Sedimentary records span the last ∼180 years in Lake Greifen, Lake Baldegg, Lake Zug, and Lake Zurich, and the last ∼600 years in Lake Lucerne (Table S5, Supplementary Material). Sediment ages were determined by excess Pb-210 and Cs-137 analyses and are from Fiskal et al. (4). *rbc*L copies were determined for all three stations per lake, biomarkers and organic macromolecules were only quantified at the deepest station in each lake.

Combining data from all lakes confirms the existence of general trends in *rbc*L copies of diatoms, green algae, and vascular plants and corresponding biomarkers and organic macromolecules through time (Fig. 3). Within each OC source organism or biomolecule group, trends in relation to time overlap strongly between lakes irrespective of trophic history. The decrease in *rbc*L copy numbers with time best matches a power function (Fig. 3a), which provides a higher fit coefficient for diatoms and green algae than exponential or linear models. This best fit of a power function is also evident when lakes are analyzed individually (Figure S5a and b, Supplementary Material). By contrast, a power relationship only explains a small fraction of variation through time in comparatively more stable vascular plant *rbc*L copy numbers (Fig. 3a). Within individual lakes, vascular plant *rbc*L copy numbers decrease significantly in Lake Greifen and Lake Zug, but not in the other lakes (Figure S5c, Supplementary Material). Calculated 18S rRNA gene copy numbers of diatoms, green algae, and vascular plants based on read percentages also follow power relationships, showing temporal trends similar to those based on corresponding *rbc*L copy numbers (Figure S6, Supplementary Material).

Similar to *rbc*L copy numbers, decreases in contents of the mainly diatom-derived biomarkers C_20:5n-3_ fatty acid and diatom sterols, and the green algal biomarker C_18:2n-6_ fatty acid through time show good matches with power functions (Fig. 3b). This is also the case for total chlorophyll and proteins (Fig. 3c) and for the microalgal biomarkers chlorophyll *a*, C_15_+C_17_+C_19_ and C_14_+C_16_+C_18_ (Figure S7, Supplementary Material). By contrast, the contents of vascular plant-derived lignin and (poly)aromatics do not decrease significantly over time and do not match power functions (Fig. 3b and c). Only the largely vascular plant-derived long chain *n*-alkanes (C_27_+C_29_+C_31_; Fig. 3b) and long-chain fatty acids (C_24_+C_26_+C_28_; Figure S7, Supplementary Material) decrease significantly through time, albeit much less than their short-chain microalgal equivalents, and match power functions.

Correlation analyses show significant relationships between diatom and green algal *rbc*L copy numbers and contents of all algal compounds (Pearson correlation, *P* < 0.001; Table S2 and Figure S8, Supplementary Material). By contrast, perhaps owing to the much lower variation in copy numbers and biomolecule contents, vascular plant *rbc*L copy numbers are not significantly correlated (*P* > 0.05) with other biomolecules, not even with other vascular plant-derived compounds.

### Organism-specific variations in biomolecule degradation rates and half-lives

**Fig. 4.**
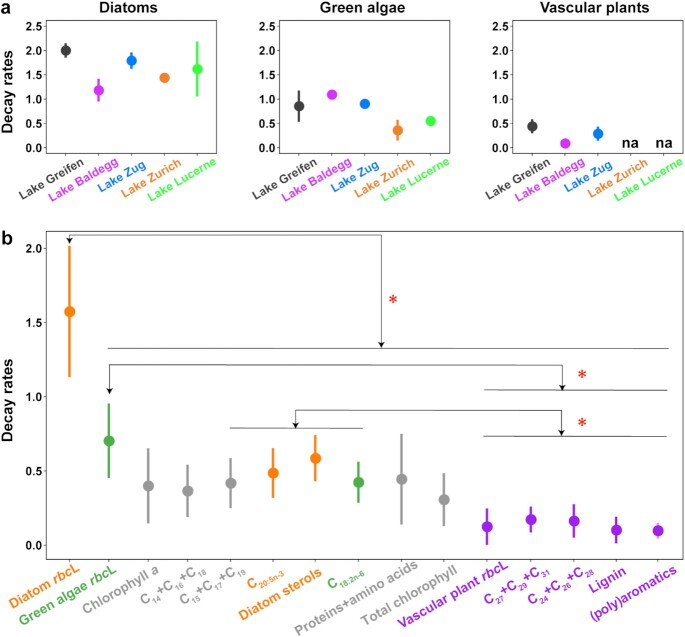
(a) Lake-specific average decay constants of *rbc*L of diatoms, green algae, and vascular plants. (b) Average decay constants of *rbc*L, biomarkers, and organic macromolecules based on lake-specific averages. Decay constants correspond to average slopes (exponents) of power functions. Error bars indicate standard deviations. Averages and standard deviations were calculated based on slope values of (a) each station per lake (*n* = 3) or (b) the deepest station in each lake (*n* = 5; note: biomarkers and macromolecules were only measured at this station). In (a) ‘na’ indicates ‘not applicable’ because *rbc*L copy numbers did not decrease over time. Colors in (b) indicate source organisms (orange: diatoms; green: green algae; purple: vascular plants; and gray: diverse microalgae). Significant differences in decay constants between biomolecules were determined using a Wilcoxon Pairwise Rank Sum Test (* = *P* < 0.05; only observed in (b)).

The exponents of the power functions in Fig. 3 and Figure S7 (Supplementary Material) indicate the decay constants of different biomolecules. Next, we compare lake-specific decay constants of *rbc*L (Fig. 4a), and average decay constants of *rbc*L, biomarkers, and macromolecules across all lakes (Fig. 4b) to provide insights into the degradation dynamics of different biomolecules.

We observe no significant effect of lake or lake trophic state on decay constants of diatom, green algae, or vascular plant *rbc*L copy numbers (Fig. 4a; *P* > 0.05, Wilcoxon Pairwise Rank Sum Test, which was used for all statistical tests in this section). However, diatom *rbc*L decay constants are consistently higher than those of green algae, whereas vascular plant *rbc*L always have the lowest decay constants.

A comparison across different biomolecules reveals that decay constants of all diatom and green algae-specific biomolecules, and all general microalgal biomolecules, are significantly higher than those of vascular plant biomolecules (Fig. 4b). Decay constants of diatom *rbc*L are, moreover, significantly (*P* < 0.05) higher than those of all other microalgal biomolecules, including diatom biomarkers (C_20:5n-3_, diatom sterols). By contrast, decay constants of green algal *rbc*L do not differ from those of the green algal biomarker C_18:2n-6_ (*P* > 0.05). Similarly, decay constants of vascular plant *rbc*L are not significantly different (*P* > 0.05) from those of corresponding vascular plant biomarkers (C_24_+C_26_+C_28_, C_27_+C_29_+C_31_, lignin) or macromolecules ((poly)aromatics).

A comparison to 18S data suggests that average group-specific *rbc*L decay constants based on gene copy numbers are comparable to decay constants of the same groups predicted based on 18S rRNA gene-based abundance estimates. The latter were calculated by multiplying group-specific 18S rRNA gene relative abundances with total 18S rRNA gene copy numbers (Figure S6b, Supplementary Material; diatoms: *rbc*L: 1.57 ± 0.44 vs. 18S: 1.82 ± 0.81; green algae: *rbc*L: 0.70 ± 0.25 vs. 18S: 0.57 ± 0.11; vascular plants: *rbc*L: 0.12 ± 0.12 vs. 18S: 0.22 ± 0.19). Thus, despite potential PCR primer biases, calculated decay constants are reproducible across *rbc*L and 18S rRNA gene-based abundance estimates.

Based on the decay constants in Fig. 4(b), we calculated biomolecule half-lives (Table 1; Figure S9, Supplementary Material). Half-lives of biomolecules from diatoms and green algae, and microalgae in general, increase linearly with sediment age, typically from a few years in 1-y-old sediment layers to millennia in 1,000-y-old sediment layers. Due to the minimum decay of vascular plant biomolecules, estimated half-lives are already thousands of years in 1-y-old sediment and millions of years in 1,000-y-old sediments (also see next section). The only exceptions are long-chain fatty acids and *n*-alkanes, which degrade over shorter time scales, though still much more slowly than biomolecules of microalgae.

**Table 1.** Half-life estimates of biomolecules from diatom, green algae, diverse microalgae (probably mainly phytoplankton), and vascular plant in relation to sediment age. Half-lives of biomolecules with an asterisk are based on power relationships with nonsignificant slope values (*P* > 0.05) and/or low coefficients of determination (*R*^2^ < 0.2).

		Half-life (years)						
	Sediment age (years)	1	20	50	100	200	500	1,000
**Diatoms**	Diatom *rbc*L	0.77	15.4	38.5	77	154	38.5	777
	Fatty acid C_20:5n-3_	2.82	56.4	141	282	564	1,410	2,820
	Diatom sterols	2.14	42.8	107	214	428	1,070	2,140
**Green algae**	Green algae *rbc*L	2.06	41.2	103	206	412	1,030	2,060
	C_18:2n-6_	3.62	72.4	181	362	724	1,810	3,620
**Microalgae**	Chlorophyll *a*	3.80	76.0	190	380	760	1,900	3,800
	Fatty acids C_14_+C_16_+C_18_	4.63	92.6	231.5	463	926	2,315	4,630
	*n-*alkanes C_15_+C_17_+C_19_	4.22	84.4	211	422	844	2,110	4,220
	Total chlorophyll	6.63	132.6	331.5	663	1,326	3,315	6,630
	Proteins	2.17	43.4	86.8	217	434	868	2,170
**Vascular plants**	Vascular plant *rbc*L*	3615	7.23E4	1.80E5	3.61E5	7.23E5	1.80E6	3.61E6
	Lignin*	3883	7.76E4	1.94E5	3.88E5	7.76E5	1.94E6	3.88E6
	*n-*alkanes C_27_+C_29_+C_31_	28.4	568	1.42E3	2.84E3	5.68E3	4.12E4	2.84E4
	Fatty acids C_24_+C_26_+C_28_	17.2	344	860	1.72E3	3.44E4	8.6E3	1.72E4
	(poly)aromatics*	1.99E4	3.99E5	9.98E5	1.99E6	3.99E6	9.98E6	1.99E7

### OC source organisms based on *rbc*L and 18S rRNA gene sequences

The observed clear differences in degradation rates between diatoms, green algae, and vascular plant biomolecules raise questions regarding the origins and sources of these biomolecules. In the case of vascular plants, the question of origin (aquatic, riparian/semi-aquatic, and terrestrial) is particularly important because terrestrial vascular plant OC can be already highly degraded (“pre-aged”) upon entry into lakes, and thus have different degradational properties than typically more “fresh” aquatic or semi-aquatic vascular plant matter. Similarly, cell wall chemical compositions vary considerably between different plant, diatom, and green algae taxa, and may affect biomolecule preservation potential among these taxa. We, therefore, perform phylogenetic analyses of *rbc*L and 18S rRNA gene sequences to identify trends in the environmental and taxonomic origins of diatom, green algae, and vascular plant DNA through time and between lakes.

**Fig. 5.**
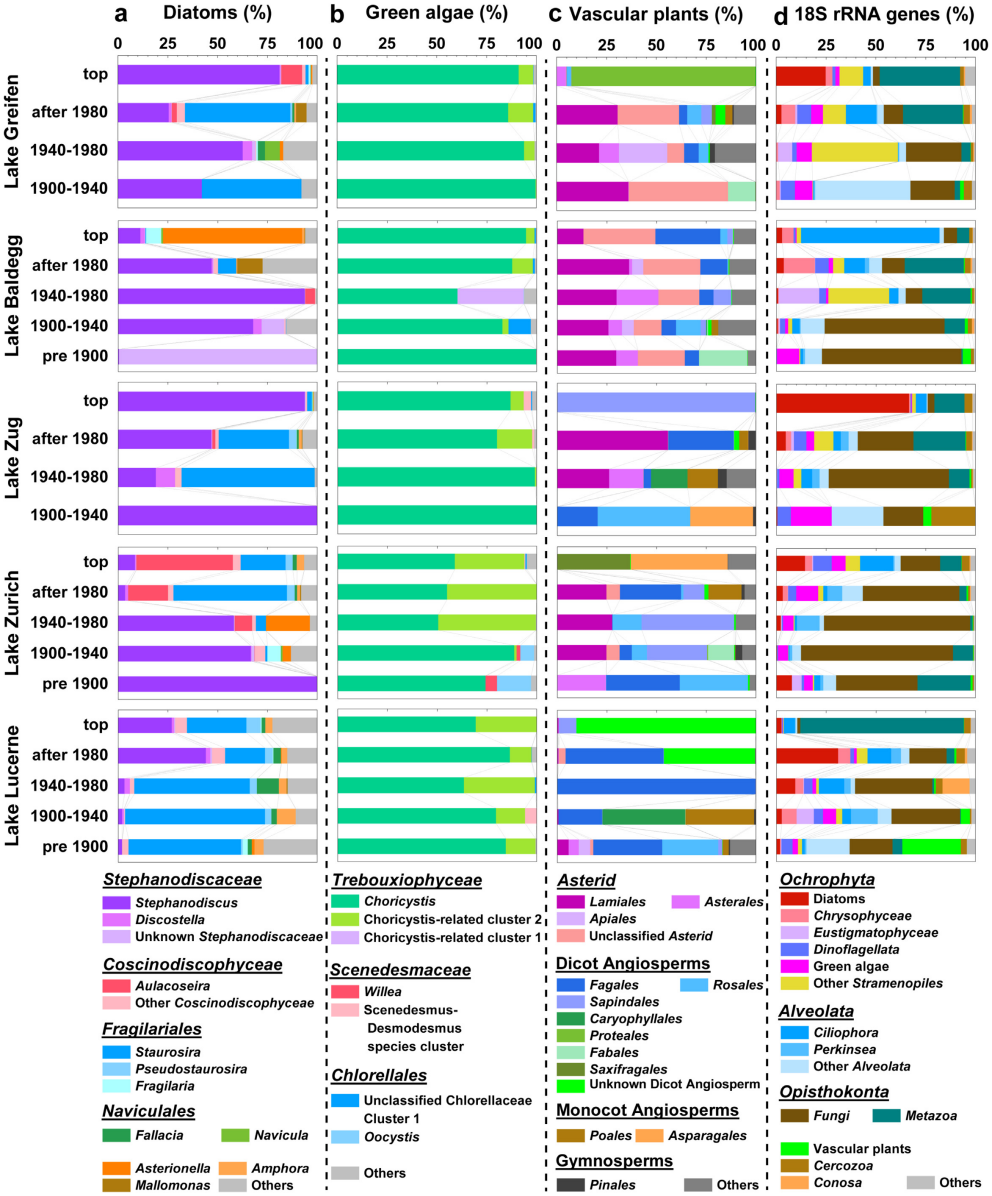
Relative abundances of (a) diatom, (b) green algae, and (c) vascular plant taxa based on *rbc*L sequences, and (d) eukaryotic taxa based on 18S rRNA gene sequences. Lakes are ordered from most eutrophic (top) to oligotrophic (bottom). Data from the sediment water-interface (top 0.5 cm, referred to as ‘top’) at the time of sampling (summer 2016) are shown separately.

The majority (on average 79 ± 21%) of diatom *rbc*L reads belong to the globally distributed diatom families *Stephanodiscaceae* (mainly *Stephanodiscus* and unknown species *Stephanodiscaceae*) and *Fragilariales* (mainly *Staurosira*; Fig. 5a and Figure S11a, Supplementary Material). Analyses of similarities (ANOSIM) show a clear impact of trophic state on diatom assemblages (*P* < 0.001; Table S3a, Supplementary Material). Matching its higher contribution to diatom *rbc*L gene sequences in samples that were deposited under eutrophic relative to oligotrophic conditions (*P* < 0.001; Table S3b, Supplementary Material), the centric, planktonic *Stephanodiscaceae* include many eutrophication indicator species that prefer high dissolved phosphorus concentrations (47). By contrast, members of *Fragilariales*, also known as “fragilarioids” due to their similar morphologies that often result in misidentifications (48), consist of pennate benthic or tychoplanktonic diatoms (49). The dominant genus *Staurosira* accounts for a higher fraction of *rbc*L reads in sediments that were deposited under oligotrophic compared to eutrophic conditions (*P* < 0.001), matching the preference of most fragilarioids for low phosphorus concentrations (47). Neither *Stephanodiscaceae* nor *Fragilariales* show significant relationships with sediment age (Table S3b, Supplementary Material). A clear lake-specific clustering of diatom communities at the ZOTU-level is also absent (Figure S10a, Supplementary Material).

Green algal DNA sequences are less phylogenetically diverse than those of diatoms and dominated by the genus *Choricystis* (class: *Trebouxiophyceae*) across all lakes (82% ± 23%; Fig. 5b and Figure S11b, Supplementary Material). This genus consists of solitary, coccoid picoplanktonic, and in some cases animal-associated cells, and is globally widespread in freshwater and brackish ecosystems of variable trophic status (50). In addition, an unknown sister lineage of *Choricystis* (*Choricystis*-related cluster 2) is abundant in oligotrophic Lake Lucerne and sediments that were deposited after 1980 in Lake Zurich. Green algal communities differ between lakes and sediment layers, but do not display lake-specific community clusters at the ZOTU-level (Figure S10b, Supplementary Material) and are not significantly correlated to trophic state (Tables S3a and S4, Supplementary Material). Percentages of *Choricystis* correlate weakly positively with sediment age (*R*^2^ = 0.11, *P* < 0.05) while those of the *Choricystis*-related cluster 2 correlate negatively with sediment age (*R*^2^ = 0.26; *P* < 0.001; Table S3b, Supplementary Material).

Vascular plant sequences are largely of terrestrial origin (Fig. 5c and Figure S11c, Supplementary Material). While community compositions differ between lakes (Figure S10c, Supplementary Material), they do not show taxon-specific trends in relation to sediment age (Table S3b, Supplementary Material) and only show minor correlations with trophic state (Tables S3a and S4, Supplementary Material). Matching the compositions of lignin phenols, most *rbc*L reads are from nonwoody plants, though decreases in ratios of p-coumaric acid+ferulic acid to vanillyl phenols (C/V) with sediment depth suggest a relative increase in woody plant tissue with sediment depth (Figure S12, Supplementary Material). Ratios of syringyl phenols to vanillyl phenols (S/V; also Figure S12, Supplementary Material), moreover, match the fact that angiosperms, i.e. mainly asterids, dicot and monocot angiosperms, account for much higher *rbc*L read percentages (80% to 100%) than gymnosperms (*Pinales*; ≤ 5%) in all samples. *Asterids* (mainly *Lamiales* and *Asterales*) dominate read fractions in Lake Greifen, Lake Baldegg, and Lake Zug, whereas dicots (mainly *Fagales* within *Betulaceae*) dominate Lake Lucerne. Dicots belonging to *Fagales, Sapindales, Rosales, Caryophyllales*, and *Fabales* are also abundant in the other lakes, as are monocot angiosperms (*Poales* and *Asparagales*) in Lake Zug, Lake Zurich, and Lake Lucerne. Despite their presence in littoral zones of all lakes, aquatic macrophytes (mainly emergent *Nymphaeales*) are weakly represented (Lake Greifen: 0.95%; Lake Lucerne: 1.7%). Potentially riparian or semi-aquatic grasses (*Poales*; unclassified *Poaceae*: on average 3.7%) and trees (*Alnus:* on average 4.4%; unclassified *Betulaceae*: on average 11.5%) are more strongly represented, especially in Lake Lucerne, where intact leaves of *Alnus* were even found in cores. Nonetheless, most vascular plant DNA is of true terrestrial origin.

18S rRNA gene sequences show a high diversity of eukaryotes (Fig. 5d and Figure S11d, Supplementary Material). Nonphotosynthetic groups are dominant with *Fungi, Metazoa*, and the SAR Supergroup contributing on average 29 ± 21%, 29 ± 23%, and 10 ± 14%, respectively, of total reads. Photosynthetic groups together account for on average 26% ± 16% of total 18S reads (diatoms: 7 ± 13%; golden algae: 3.4 ± 4.5%; *Eustigmatophyceae*: 1.8 ± 4.1%; green algae: 5.1 ± 4.7%; and vascular plants: 2.9 ± 9.5%). There are no clear lake-specific 18S rRNA gene community clusters (Figure S10d, Supplementary Material) or statistically significant differences in total eukaryotic communities in relation to trophic state (Tables S3a and S4, Supplementary Material). While contributions of diatoms (*P* < 0.01), golden algae (*P* < 0.05), *Ciliophora* (*P* < 0.01), and *Metazoa* (*P* < 0.01) decrease significantly with sediment age, contributions of green algae do not change significantly with sediment age. Moreover, contributions of *Fungi* (*P* < 0.01) and especially vascular plants (*P* < 0.001) increase significantly with sediment age (Table S3b, Supplementary Material).

## Discussion

### Postburial processes primarily control microalgal biomolecule inventories

By analyzing DNA, biomarker, and organic macromolecule records through time, thereby comparing organic compounds with widely differing chemical reactivities, we provide novel insights into the controls on long-term OC preservation in lake sediments. We show that the degradation of microalgal DNA, lipid biomarkers, and organic macromolecules follows a power relationship, and that sediment age profiles and inferred degradation rates of these biomolecules are in the same range across lakes with different trophic histories (Figs 3 and 4a). This indicates that—despite the significant influence of trophic state on OC inputs and long-term burial in the lakes studied (4)—postburial processes are more important in determining long-term microalgal biomolecule inventories than past trophic state. Previous studies have shown that total OC content follows a power relationship with sediment age in marine and lacustrine sediments (4, 43), and that inventories of amino acids, carbohydrates, and lipids over time follow first-order kinetic power models in marine sediments (51–53). We demonstrate that power functions also describe lake sedimentary inventories of DNA, biomarkers, and organic macromolecules of individual groups of microalgae (diatoms and green algae) and of microalgae as a whole (Fig. 3 and Figure S7, Supplementary Material).

Relationships between vascular plant-derived OC and sediment age differ from those in microalgae. Long-chain *n-*alkane and fatty acid contents decrease significantly through time, albeit much more slowly than short-chain *n-*alkane and fatty acids of microalgal origin, and follow power-relationships (Fig. 3 and Figure S7, Supplementary Material). By contrast, inventories of DNA, lignin, and (poly)aromatic compounds do not decrease significantly through time or follow power relationships. In the following sections, we first analyze and interpret the observed differences in DNA degradation rates between diatoms, green algae, and vascular plants based on the framework of Fig. 1. We then offer explanations for the observed degradation trends in lipids and organic macromolecules.

### Controls on the long-term preservation of DNA

Age profiles of DNA differ significantly between microalgae and vascular plants, and also between the two microalgal groups, suggesting differences in degradation controls between diatoms, green algae, and vascular plants. Consistent with past evidence that membrane lipids are more resistant to enzymatic degradation than DNA (39), DNA of diatoms degrades significantly faster than the mainly diatom-derived biomarkers C_20:5n-3_ and diatom sterols (Figs 3 and 4b). This suggests that degradation rates of diatom biomolecules are strongly influenced by chemical reactivity in the lakes studied (Fig. 1). This is not the case for green algal DNA, however, which degrades significantly (two to three times) slower than diatom DNA, at a rate that does not significantly differ from that of the chemically less reactive green algal biomarker C_18:2n-6_ (Fig. 4b). Vascular plant DNA, which is mainly of terrestrial origin, even remains stable over centuries, similar to lignin, (poly)aromatics, long-chain fatty acids and *n-*alkanes, which are all highly degradation resistant (54). The fact that, based on quantifications of diagnostic genes, labile DNA of green algae and vascular plants shows similar relationships with sediment age as chemically more resistant biomolecules from the same organisms indicates that factors besides chemical reactivity must control the degradation of this DNA.

Adsorption to mineral matrices by electrostatic interactions (36, 37) and complexation with organic and inorganic substances (38) have been proposed to shelter extracellular DNA, e.g. released by cell lysis, from nuclease attack. Yet, several studies indicate that extracellular DNA is degraded over time scales of weeks to years (31, 55, 56), which would indicate that any protection by adsorption or complexation is not long-term. Based on the framework in Fig. 1, our results also argue against a major role of adsorption or organic complexation. Due to its chemical equivalency, DNA of diatoms, green algae, and vascular plants would expectedly act similarly in terms of its adsorption and complexation behavior, and thus be degraded at similar rates. Instead we observe clear differences in degradation rates between all three groups (Fig. 4b), with calculated DNA half-lives differing by three orders of magnitude between diatoms and vascular plants (Table 1).

Biotic exclusion by physical shielding inside dead or buried cells with intact cell walls offers an alternative explanation for the slower degradation rates of green algal and vascular plant DNA. Outer cell walls of many green algae contain the recalcitrant, chemically poorly characterized polyester heteropolymer algaenan, which effectively resists biological (viral, enzymatic, and grazing) attack and chemical extraction (57, 58, and references therein). Experiments with the green algal species *Botryococcus braunii* have indicated that greater resistance of algaenan-rich cell walls to biological attack leads to ∼8-fold slower degradation of intracellular proteins in *B. braunii* compared to cyanobacteria, diatoms, and dinoflagellates (59). This helps explain the preservation of algaenan in the fossil record, and the significant contribution of algaenan to kerogen (60). The dominant green algae in this study cluster with coccoid picoplanktonic *Choricystis*, which form a monophyletic group with *Botryococcus* in the class *Trebouxiophyceae* (61). Thus, the significantly lower degradation rates of green algal compared to diatom DNA could be due to intracellular protection by algaenan-containing cell walls. Our observation that green algal DNA is selectively preserved over DNA of other algae is in line with past studies showing increasing genetic contributions of green algal *Trebouxiophyceae* with sediment depth and age in Holocene marine sediments (62, 63).

Encapsulative shielding of DNA can also explain the absence of significant degradation of vascular plant DNA. Recalcitrant biomolecules that make up large portions of plant cell walls and woody tissue, such as lignin (54), could shield DNA inside these structures over time scales of centuries and longer. Past research has shown that decomposition rates of vascular biomass are negatively correlated with lignin content, which is generally highest in terrestrial and lowest in aquatic plants (64–66, and references within). Hereby lignin provides a physical barrier to extracellular enzymes and shields cellulose and other plant cell components from enzymatic digestion (67, 68). The clear dominance of true terrestrial, and to a lesser degree riparian or semi-aquatic, vascular plant over aquatic vascular plant DNA sequences, despite the widespread occurrence of aquatic plants in littoral zones of all five lakes (Fig. 5), is consistent with an important role of lignin. We, thus propose that the high lignin contents of terrestrial plant cells contribute to the selective preservation and predominance of terrestrial plant DNA in sediments of this study.

### Controls on the long-term preservation of lipids and other macromolecules

The observed trends in biomarker and organic macromolecule inventories are more difficult to interpret than those of DNA. This is especially the case for phytoplanktonic (microalgal and cyanobacterial) biomarkers and macromolecules, which strongly overlap in degradation rates, and are not entirely specific to any group of microalgae (10, 14). The latter issue adds a degree of uncertainty to the interpretation of microalgal biomarkers especially for older sediment layers. For instance, it is not certain that C_20:5n-3_ and ‘diatom sterols’, which are enriched in, but not unique to, diatoms, in older, highly diagenetically processed sediment layers are still predominantly of diatomaceous origin. Nonetheless, several general inferences can be made.

In contrast to reports that microalgal lipid degradation follows chemical reactivities, thus decreasing from fatty acids to sterols to *n*-alkanes and from unsaturated to saturated fatty acids (22), we observe no relationship between reactivity and microalgal lipid degradation rates. Short-chain lipids, independent of whether they are saturated (C_14_+C_16_+C_18_) or polyunsaturated fatty acids (C_20:5n-3_, C_18:2n-6_), sterols (diatom sterols), or *n*-alkanes (C_15_+C_17_+C_19_) do not differ significantly from each other in their decay constants (Fig. 4b). This matches reported discrepancies between chemical reactivity and inferred lipid degradation rates in coastal sediment (69). We, moreover, observe that decay constants of microalgal membrane lipids are in the same range as those of other, more reactive compounds of microalgal origin, such as chlorophyll *a*, total chlorophyll, and proteins. Collectively, these trends imply that chemical reactivity is not the sole or main driver of microalgal biomolecule degradation rates in the lakes studied. Based on our insights from DNA pools, we postulate that—at least in certain microalgae—biotic exclusion by recalcitrant cell walls plays a significant role in the long-term preservation of lipids, chlorophylls, and proteins.

Unlike microalgal biomolecules, vascular plant-derived biomolecules in the sediments studied appear to be of mainly terrestrial origin (Fig. 5). Thus, most vascular plant organic matter is likely to have already been significantly degraded (“pre-aged”) before deposition to lake sediments. While this difference in the initial stage of degradation at the sediment surface prohibits general comparisons of microalgal and vascular plant biomass degradation rates, the measured age profiles nonetheless offer insights into the sources and preservation mechanisms of this preaged terrestrial plant matter.

Previous studies have indicated an important role of mineral protection, e.g. by occlusion within inaccessible pore space or formation of bonds to solid-phase minerals, in the preservation of terrestrial biomolecules (35, 41). This mineral protection, which is important in soils and during fluvial transport and deposition to sediments, can effectively preserve terrestrial plant long-chain *n*-alkanes and fatty acids, which primarily derive from leaf waxes, for thousands of years (18, 70). It is possible that most long-chain *n*-alkanes and fatty acids in the sediments studied were transported and subsequently protected in sediments via associations with soil minerals. If so, then biotic exclusion by mineral associations could explain the much lower degradation rates of mainly plant-derived long-chain *n*-alkanes and fatty acids compared to their short-chain, mainly microalgal counterparts.

Unlike the frequently mineral-associated long-chain *n*-alkanes and fatty acids, which are from leaf cuticles of plants, sedimentary lignin, which is a dominant component of plant cell walls and fibers, is primarily introduced by surface runoff of plant detritus (18). Such plant detritus can account for a significant portion of terrestrial plant matter in sediments (71). The absence of apparent lignin degradation over time in the lakes studied (Fig. 3) is striking and indicates that lignin-rich, preaged terrestrial plant detritus is only minimally degraded after burial. Even more remarkable is the apparent lack of vascular plant DNA degradation through time. We interpret this absence of clear DNA and lignin degradation as a potential indication that the fate of both biomolecules is coupled. We hypothesize that the main protection mechanism of terrestrial plant DNA in sediments of the lakes studied is encapsulation by recalcitrant, lignin-rich cell wall components. Our interpretation is supported by past observations of long-term preservation of chemically intact DNA in detrital plant tissue (29, 72, 73) and research showing that lignin-rich cell walls effectively block extracellular enzymes and shield cellulose and other plant cell molecules from enzymatic digestion (67, 68).

### Pertinence of findings to other sedimentary settings

Our analyses suggest a key role of biotic exclusion in the long-term preservation of sedimentary biomolecules in the lakes studied, but raise questions regarding the extent to which the observed insights are transferrable to other locations. While the dominant diatom, green algae, and vascular plant taxa in our study are globally widespread in aquatic and terrestrial settings, they do not ubiquitously dominate freshwater and terrestrial, let alone marine settings. We postulate that biotic exclusion (physical shielding) by recalcitrant cell walls is an important biomolecule preservation mechanism also in other sedimentary settings, but that organism group-specific trends observed in our study do not apply to (all) other locations. Instead the importance of biotic exclusion by recalcitrant cell walls may vary with the taxonomic composition of diatoms, green algae, vascular plants, and other organisms.

Though chemical processes (74) and bacteria (75) can dissolve diatom silica, microfossils, and DNA of the thick-walled, cyst- and spore-forming diatom genus *Chaetoceros*, which was not detected here, are selectively preserved over other diatom taxa in marine sediments, and detectable over time scales of millennia (47, 60) or longer (51). This indicates that degradation of diatom biomass is not universally driven by chemical reactivity. Instead, in locations where diatom groups with highly silicified cell walls and/or resting stages are dominant, degradation of diatom biomolecules could be primarily controlled by the same physical shielding mechanisms inferred for green algae and terrestrial vascular plants in this study. Similarly, the degradation controls of green algal biomass may vary with taxonomic compositions of green algae, since many green algal groups lack algaenan (76). Moreover, as discussed earlier, it is known that vascular plant biomass varies greatly in biodegradability as a function of lignin content, which varies in relation to habitat, tissue type, as well as taxonomic group (64, 65). Collectively, these differences in biodegradability among organism groups underscore the importance of knowing the identities of organic matter source organisms and with that the great value of DNA-based community analyses as a tool to understand and predict the burial of OC in any particular setting.

## Conclusions

Based on the results of our study, we propose that biotic exclusion by recalcitrant cell walls exerts a key influence on the preservation of biomolecules from certain organism groups in lake sediments. Hereby, protection by recalcitrant cell walls may explain why even labile intracellular biomolecules, such as DNA polymers, remain intact and stable in content over centuries and longer. Our results furthermore raise questions regarding the contributions of different organisms to long-term burial of OC in lacustrine and other sediments. Despite representing a dominant OC source in surface sediments, diatom biomolecules in the lakes studied are more rapidly degraded than those of green algae and vascular plants and may, therefore, account for lower fractions of buried OC in older sediment layers than the other groups.

To assess the general importance of recalcitrant cell walls in driving OC burial in sediments, further research across diverse spatial and temporal scales is needed. Laboratory degradation experiments involving pure cultures of organisms that differ in cell wall compositions represent a crucial means toward demonstrating relationships between cell wall chemical compositions, cell wall integrity, and biomolecule preservation through time. In addition, the analysis of eukaryotic cells and multicellular structures from sedimentary sequences that span geologic time scales is essential for understanding temporal processes that cannot be replicated in the laboratory. A key to understanding lies in combining quantitative analyses on bulk samples with microscale observations and measurements on single cells and multicellular structures, e.g. microspectroscopic analyses of cells and cell wall chemical compositions combined with DNA sequencing. Such integrations of large-scale quantitative research with mechanistic studies at the microscale, where most OC transformations take place, are crucial to understanding the fate of sedimentary OC and to identifying the contributions of different organisms to long-term sedimentary OC burial.

## Materials and Methods

### Background and environmental context

This study is part of the research effort “Lake Eutrophication Impacts on Carbon Accumulations in Sediments” (LEICAS; https://www.researchgate.net/pr oject/Lake-Eutrophication-Impacts-on-Carbon-Accumulation-in-Sediments-LEICAS), in which the long-term impacts of eutrophication on the biogeochemistry, microbiology, and ecology of lake sediments is investigated. LEICAS was started in 2016, when sediments of Lakes Lucerne, Zurich, Zug, Baldegg, and Greifen in central Switzerland were sampled at three stations that ranged from shallow sublittoral to profundal (Figure S13 and Table S5, Supplementary Material). Biogeochemical, organic geochemical, and microbiological abundance and community data on all stations were published previously (4, 9, 77).

The five lakes differ in trophic history. Lake Greifen, Lake Baldegg, Lake Zug, and Lake Zurich experienced severe anthropogenic eutrophication—manifested through algal blooms, and anoxic events, and driven by increased anthropogenic input of P—starting in the late 19th or early 20th century (Lake Greifen: ∼1920; Lake Baldegg: ∼1870; Lake Zug: ∼1930; and Lake Zurich: ∼1890) (4). As a result, these lakes became highly eutrophic from ∼1950 to 1980, whereas Lake Lucerne remained oligotrophic and only experienced slight increases in water column P concentrations during the mid 20th century. Since the 1970s, advanced wastewater treatment plants, P bans on detergents, and changes in agricultural practices have strongly decreased P inputs to all lakes. Yet, while Lake Zurich has since become mesotrophic, Lake Greifen, Lake Baldegg, and Lake Zug have remained eutrophic. Phosphorus release from sediments into overlying water is believed to sustain high rates of primary production (4) and explain why microalgal biomass, including that of diatoms and green algae, has not decreased in Lakes Greifen, Baldegg, or Zurich since the era of peak eutrophication (78–80; no monitoring data for Lake Zug). Instead, microalgal biomass in oligotrophic Lake Lucerne has for unknown reasons dropped 3-fold in recent years (from ∼30 g m^–2^ from 1961 to 1998 to ≤ 10 g m^–2^ after 2002 (81).

### Sample collection

Sampling took place in June and July 2016. The top ∼40 cm of sediment were recovered using 150-mm diameter gravity corers with clear plastic liners (UWITEC, Austria). In each lake, three stations (shallow, medium, and deep) from the shallow sublittoral to profundal zone were sampled (for depth intervals and ages see Table S6 (Supplementary Material)). Distances to shore ranged from tens of meters (shallow sublittoral stations) to ∼0.5 to 1.5 km (profundal sites). After each station, cores were transferred to shore, where they were vertically extruded and sliced using a cutting plate. Sediments for DNA analyses (∼20 samples per core) were sampled using sterile, cut-off 3-ml disposable syringes and immediately transferred to sterile 5-ml cryotubes, flash-frozen in liquid nitrogen, and thereafter stored at −80°C. Sediments for biomarker and Py–GC/MS analyses were taken from the same core right after DNA sampling using clean metal spatulas, stored on ice in Whirlpak bags during sampling, and thereafter frozen at −20°C.

### OC measurements and degradation rate modeling

Lipid biomarkers (*n*-alkanes, saturated fatty acids and sterols), except the polyunsaturated fatty acids C_20:5n-3_ and C_18:2n-6_, and lignin phenol data were published in Han et al. (9). C_20:5n-3_ fatty acid and ‘diatom sterols’ [brassicasterol (24-methylcholesta-5,22E-dien-3β-ol) + 24-methylenecholesterol (24-methylcholesta-5,24(28)-dien-3β-ol) were used as biomarkers for diatoms (13), whereas C_18:2n-6_ fatty acid was used as a biomarker of green algae (10). OC macromolecular composition was determined by Py–GC/MS as described previously (20); also see Supplementary Text). Biomolecule degradation through time was modeled by fitting a power function based on Middelburg (43). In this power function, Y = *a* · X*^b^*, Y equals biomolecule content (or gene copies) g^–1^ dry sediment, and X is sediment age in years. *a* and *b* are constants, where *b* is the slope of the power function, and thus the first-order decay rate (referred to as ‘decay constant’ throughout the manuscript). Biomolecule half-lives were calculated based on the power function of biomolecule content (or gene copies) vs. sediment age. Because biomolecule reactivity decreases as a function of time according to the power function, calculated half-lives are not constant but increase with sediment age (Figure S9, Supplementary Material).

### DNA extraction

DNA was extracted according to Lever et al. (82). Sediments from Lake Zug, Lake Zurich, and Lake Lucerne were extracted with lysis protocol II while those from the eutrophic Lake Greifen and Lake Baldegg underwent an additional humic acid removal step (lysis protocol III), which did not impact DNA sequence compositions (9). For the exact protocol, see Han et al. (9).

### Quantification and sequencing of eukaryotic 18S rRNA genes and *rbc*L

To investigate vertical changes in the abundances of eukaryotic 18S rRNA genes and *rbc*L (diatom, green algae, and vascular plants), qPCR (∼20 depths per deep station, ∼10 depths from shallow and medium station), using SYBR Green I Master on a LightCycler 480 II (Roche Molecular Systems, Inc.). Details on qPCR primers, sequences, and standards are shown in Table 2. While we were able to identify a high-coverage and specific published primer pair for vascular plant *rbc*L qPCR assays and Illumina Paired-End sequencing, it was necessary to design new group-specific *rbc*L primer combinations for diatoms and green algae to meet the same criteria (Table S7, Supplementary Material).

**Table 2.** PCR primer target groups, names, sequences, references, annealing temperatures, and product length.

Targeted groups	Primers	Sequence (5´-)	Reference	Annealing T (°C)	Product length (∼bp)
All eukaryotes (18S rRNA gene)	All18S F_mod1	TGC ATG GCC GTT CTT AGT	(62, 63)	55	170
	All18S R_mod1	CTA AGG GCA TCA CAG ACC			
Vascular plants (*rbc*L)	*rbc*L h1aF	GGC AGC ATT CCG AGT AAC TCC TC	(84)	55	130
	*rbc*L h2aR	CGT CCT TTG TAA CGA TCA AG			
*Ochrophyta* (*rbc*L)	Ochro-rbcL_43F	CGT TAC GAA TCT GGT GTA AT	This study	55	390
	Ochro-rbcL_432R	GGA ATA CGC ATA TCT TCT AAA CGT A			
*Chlorophyta* (*rbc*L)	Chloro-rbcL_110F	TWG CRG CWT TYC GTA TGA CIC	This study	54	430
	Chloro-rbcL_537R	CCT AAK TTW GGT TTA ATN GTA CA			

The same 18S rRNA gene and *rbc*L primer pairs used for qPCR were used for amplicon sequencing using a MiSeq Personal Sequencer (Illumina Inc., San Diego, California, USA). A total of 11 depths for *rbc*L and seven to nine depths for 18S rRNA genes (same samples as for Bacteria and Archaea in Han et al. (9)), in each case covering the entire cored sediment histories, were chosen from the deep stations. For details on library preparation and sequencing, see Han et al. (9) and Supplementary Text.

### Sequencing data processing

Sequences were processed according to Han et al. (9), with raw reads first quality-checked, read ends trimmed by seqtk (https://github.com/lh3/seqtk), and reads merged into amplicons by flash (max mismatches density, 0.15). Primer sites were trimmed by usearch (in-silico PCR). Quality filtering was done by prinseq (GC range, 30 to 70; Min Q mean, 20). Zero-radius operational taxonomic unit (ZOTU), used as a proxy for eukaryotic species, were generated using the USEARCH unoise3() algorithm with a 99% identity, which includes the removal of chimeric sequences. A total of 3,220 ZOTUs for 18S rRNA gene (6,092,879 reads), 537 ZOTUs for *Ochrophyta* (2,997,071 reads), 75 ZOTUs for *Chlorophyta* (1,516,590 reads), and 82 ZOTUs for vascular plants (4,988,703 reads) were detected, respectively. ZOTU count tables were generated by USEARCH otutab(). 18S rRNA ZOTUs were taxonomically assigned using the Protist Ribosomal Reference database (PR2 v.19 (83), confidence threshold: 0.9). *rbc*L sequences of *Ochrophyta, Chlorophyta*, and vascular plants were taxonomically assigned based on *de novo* phylogenetic trees that were based on a new database that was constructed in ARB and consisted of ∼1,400 bp long-read *rbc*L sequences with manually optimized sequence alignments (http://www.arb-home.de). All phylogenetic trees were built by neighbor-joining using Jukes-Cantor correction. Since diatoms (*Bacillariophyta*) accounted for > 98% of recovered *Ochrophyta* sequences from the lakes studied, we only focus on diatom sequences.

## Supplementary Material

pgac076_Supplemental_File

## Data Availability

All raw sequences of *rbc*L and 18S rRNA genes are publicly accessible at the National Center for Biotechnology Information (NCBI; SAMN13038023, project PRJNA577818).
