# Supplementary material for: Long-term preservation of biomolecules in lake sediments: potential importance of physical shielding by recalcitrant cell walls
Source: PNAS Nexus. 2022 Jun 8;1(3):pgac076. doi: 10.1093/pnasnexus/pgac076 (PMC9896894; doi:10.1093/pnasnexus/pgac076)
Supplement: pgac076_Supplemental_File [file pgac076_supplemental_file.docx]

**Supplementary Materials**

**Long-term preservation of biomolecules in lake sediments: potential importance of physical shielding by recalcitrant cell walls**

Xingguo Han^a,†,*^, Julie Tolu^a,b^, Longhui Deng^a^, Annika Fiskal^a,§^, Carsten Johnny Schubert^a,c^, Lenny H.E. Winkel^a,b^, Mark Alexander Lever^a,*^

a. Institute of Biogeochemistry and Pollutant Dynamics, Swiss Federal Institute of Technology, Zurich (ETH Zurich), Universitätstrasse 16, 8092 Zurich, Switzerland

b. Department of Water Resources and Drinking Water, Swiss Federal Institute of Aquatic Science and Technology (EAWAG), Überlandstrasse 133, 8600 Dübendorf, Switzerland

c. Department of Surface Waters - Research and Management, Swiss Federal Institute of Aquatic Science and Technology (EAWAG), Seestrasse 79, 6047 Kastanienbaum, Switzerland

†. Current address: Forest Soils and Biogeochemistry, Swiss Federal Institute for Forest, Snow and Landscape Research (WSL), Zürcherstrasse 111, 8903, Birmensdorf, Switzerland

§. Current address: Department of Microbial Ecology, German Federal Institute of Hydrology (BfG), Am Mainzer Tor 1, 56068, Koblenz, Germany

*To whom correspondence should be sent:

Xingguo Han (email: [xingguo.han@wslch](mailto:xingguo.han@wslch)), Mark Alexander Lever (email: mark.lever@usys.ethz.ch)

Institute of Biogeochemistry and Pollutant Dynamics, Swiss Federal Institute of Technology, Zurich (ETH Zurich), Universitätstrasse 16, 8092 Zurich, Switzerland

Phone: +41 44 632 85 27

Fax: +41 44 633 11 22

# **Supplementary Text**

## **Lipid Biomarkers and lignin analyses**

**Fatty acids and neutral lipids**. Fatty acids (FAs) and neutral lipids (*n*-alkanes and sterols) were extracted using a method modified from Naeher*, et al.* (1). 2-3 g of freeze-dried sediment were extracted with 15 ml of a mixture of methanol (MeOH, 4.5 ml) and dichloromethane (DCM, 10.5 ml) at 70 °C for 6 min in a Microwave Reaction System (SolvPro, Anton Paar, Graz, Austria). An internal standard (5α-cholestane, C19 n-alcohol and C19:0 fatty acid) was added. Extracts were then saponified with 6% KOH in MeOH at 80 °C for 3 h. Total neutrals were extracted (3 times) with hexane and dried under a stream of N_2_. Subsequently the neutral fractions (*n*-alkanes, sterols and stanols) were derivatized with BSTFA [N,O-bis(trimethylsilyl) trifluoroacetamide] for 15 min at 70 °C to form trimethylsilyl ethers. FAs were extracted with hexane (3 times) from the remaining fraction after acidification to pH 2, and were derivatised with 14% BF_3_/MeOH (Sigma Aldrich) for 2 h at 100 °C to generate methyl esters. The resulting methyl esters were extracted with 2 mL hexane (3 times). FAs and neutral lipids were measured using a gas chromatograph (GC) with flame ionisation detection (Shimazdu, Kyoto, Japan). Samples were injected by an AOC-20i auto-sampler (Shimadzu) through a split/splitless injector operated in splitless mode at 280 °C. An InertCap 5MS/NP GC column (0.25mm × 30m × 0.25 μm) (GL Sciences, Japan) and a temperature program of 70 to 130 °C at 20 °C min^-1^, from 130 °C to 320 °C at 4 °C min^-1^, and at 320 °C for 20 min was used. Peaks of interest were identified by standards and using a gas chromatography-mass spectrometry (QP2020 mass spectrometer (Shimadzu, Japan)) instrument under identical conditions.

Among the lipid biomarkers that were targeted, short chain *n*-alkanes (C_15_+C_17_+C_19_) and short chain fatty acids (C_14_+C_16_+C_18_) are mainly from phytoplankton and Bacteria, and long chain *n*-alkanes (C_25_+C_27_+C_29_) and long chain fatty acids (C_24_+C_26_+C_28_) from vascular plants (2-4). The sterols brassicasterol and 24-methylene chlolesterol are mainly produced by diatoms^,^ but a contribution from higher plants is also possible (5, 6). The unsaturated fatty acid C_20:5n-3_ occurs predominantly in diatoms and the unsaturated fatty acid C_18:2n-6_ mainly in green algae (*Chlorophyta*) (7, 8).

**Lignin phenols**. Lignin-derived phenols were extracted by alkaline CuO oxidation with a microwave digestion system according to the protocol described in Goñi and Montgomery (9). Freeze-dried sediment (2-5 mg of organic carbon (OC)) was reacted with ~500 mg of CuO powder, 50 mg of ferrous ammonium sulfate ((NH_4_)_2_Fe(SO_4_)_2_) and ~15 mL of N_2_-bubbled NaOH (2 M) in N_2_-pressurized Teflon vessels for 90 min at 150 °C in a Microwave Reaction System (SolvPro, Anton Paar, Graz, Austria). After the oxidation, known amounts (50 µL) of recovery standards (ethyl vanillin and *trans*-cinnamic acid) were added to each vessel. The content of each vessel was transferred to a Pyrex centrifuge tube and centrifuged to separate solid and aqueous hydrolysates. Aqueous hydrolysates were acidified with HCl (~4-5 mL) to pH 1. Oxidation products were then extracted twice with 6-8 mL ethyl acetate and dried under an N_2_ stream. Excessive water in ethyl acetate extracts was eliminated by sodium disulfate (Na_2_SO_4_), and the extract was then re-dissolved in pyridine. Prior to injection into a GC, an excess volume (20 µL) of N,O-Bis(trimethylsilyl)trifluoroacetamide (BSTFA) with 1% trimethylchlorosilane (TMCS) was added into the injection vial and derivatized at 60 °C for 30 min. An internal standard (3,4-dihydroxybenzoic acid) was added to the samples. Eight quantified products were measured: vanillyl (V) phenols (vanillin, acetovanillone, vanillic acid), syringyl (S) phenols (syringaldehyde, acetosyringone, syringic acid), and cinnamyl (C) phenols (*p*-coumaric acid, ferulic acid).

## **Organic macromolecule compositional analyses by Py-GC/MS**

Sediments from the deepest station in each lake were chosen for organic macromolecule analysis as described in Tolu, Gerber, Boily and Bindler (10). The analytical setup consisted of an oven pyrolyzer equipped with an auto sampler (PY-2020iD and AS-1020E, FrontierLabs, Japan) connected to a GC/MS system (Agilent, 7890A-5975C, Agilent Technologies AB, Sweden). Sediments were lyophilized overnight, ground and thoroughly homogenized. 500 to 1500 µg of dry sediment (prepared by weight with an XP6 Mettler-Toledo micro-balance) was pyrolyzed at 450°C using a split ratio of 6:1 (i.e., 16.7 % of the volatilized molecules in the pyrolysis oven is injected into the GC column). The aim of adjusting the sediment weight was to pyrolyze and inject a similar amount of OC across samples (total OC ranged from 1.1-4.2 dry weight %). Data were processed in R (version 2.15.2, 64 bits; <http://www.R-project.org>) with a data-processing pipeline consisting of chromatogram smoothing and alignment, background correction and multivariate curve resolution by alternate regression (details in Gerber, Eliasson, Trygg, Moritz and Sundberg (11)). Peaks were identified from mass spectra using the ‘NIST MS Search 2.00 software (<http://chemdata.nist.gov/dokuwiki/doku.php?id=chemdata:ms-search>) and the ‘NIST/EPA/NIH 2011’ library, which had been expanded with additional literature spectra reported as described in Tolu, Gerber, Boily and Bindler (10).

We focused on the pyrolytic products of proteins (plus non-protein amino acids) and total chlorophyll, both of which mainly derive from aquatic phytoplankton, and on long chain *n*-alkanes (C_27_+C_29_+C_31_) and lignin oligomers that dominate in vascular plants. A detailed list of the identified pyrolytic organic compounds for these compound families is given in Table S1, together with the compound chemical formula, molecular weight and reference for their mass spectra. To calculate decay rates and perform statistical analyses, we normalized peak areas of identified pyrolytic organic compounds to the exact mass of sediment analyzed (unit: peak area g^-1^ dry sediment). Py-GC/MS is a semi-quantitative method, i.e., the peak area of each Py compound is proportional to the concentration of the organic compounds the Py compounds derive from. This is illustrated by the fact that, as in previous studies (10, 12), the sum of peak areas of all identified pyrolytic organic compounds (105 in total) was significantly correlated with total sedimentary OC content (R^2^= 0.62; p<0.01; Fig. S1).

## **Polymerase Chain Reaction (PCR) assays for amplicon sequencing**

PCRs on eukaryotic 18S rRNA and *rbc*L genes were done in 25-µL reactions using GoTaq@ Hot Start Colorless Master Mix (Promega Corporation) with 1-2 µL template DNA, 10 mg mL^-1^ BSA and 10 µM of each primer. We performed a three-step PCR setup consisting of an initial booster PCR with regular primers, followed by subsequent PCRs using tailed, and indexed primer pairs. In each amplification step, we used the minimal number of PCR cycles required to obtain visible PCR products on an agarose gel. Booster PCRs consisted of: (1) initial denaturation at 95 °C for 5 min; (2) 16-25 cycles (equivalent to threshold cycle numbers for qPCR detection) of (a) denaturation at 95 °C for 20 s, (b) annealing at 49 °C for 30 s, and (c) elongation at 72 °C for 30 s, and (d) final elongation at 72 °C for 5 min. For tailed-primer PCRs, 1µL of PCR product from the booster PCR was amplified using the same reaction mixture (but without BSA) and thermal cycling conditions for 10 cycles. For the subsequent index PCR, 2 µL of tailed-primer PCR product was used, and the index primers (Sigma-Aldrich, Switzerland) and the same reaction mixture and thermal cycling conditions as for the tailed PCR were used for an additional 8 PCR cycles. PCR products of tailed primer PCR and index primer PCR were purified using the Agencourt AMPure XP system (Beckman Coulter) with a ratio of 0.8:1. All products from the above three PCR reactions were checked on an agarose gel using a Bio-Rad Gel Doc 2000 system. Final, indexed and purified PCR products were quantified with a Spark 10M Multimode Microplate Reader (Tecan) and pooled in equimolar amounts. The size distribution and concentration of the pooled amplicons were measured on a 2200 TapeStation with High sensitivity D1000 ScreenTape (Agilent Technologies), and also with a Qubit 2.0 Fluorometer with Qubit dsDNA BR Assay Kit (Thermo Fisher Scientific). 300 bp pair-end sequencing (600 cycles) was done at the Genetic Diversity Centre of ETH Zurich using a MiSeq Personal Sequencer (Illumina Inc.). Pooled DNA was loaded to a concentration of 10 pM containing 10 % PhiX.

# **Supplementary Tables**

# **Table S1. Identified organic compounds by Py-GC/MS used in this work along with their chemical formula, molecular mass (M), and references for the mass spectra.**

|  | | | |
| --- | --- | --- | --- |
| Name | Formula | M | Ref. for the mass spectra |
| **Proteins and non-protein amino acids** |  |  |  |
| Diketopiperazine (DKP) Pro-Val | C10H16N2O2 | 196 | (13) |
| Diketopiperazine (DKP) Leu-Pro |  | 210 | (13) |
| Diketopiperazine (DKP) Pro-Lys-NH3 |  | 208 | (13) |
| **Total chlorophyll** |  |  |  |
| Prist-1-ene | C19H38 | 266 | (14) |
| Prist-2-ene | C19H38 | 266 | (14) |
| Phytene | C20H40 | 280 | NIST |
| Phytadiene 1 | C20H38 | 278 | (14) |
| Phytol | C20H40O | 296 | NIST |
| Phytadiene 2 | C20H38 | 278 | (14) |
| Phytadiene 2 | C20H38 | 278 | (14) |
| **Long-chain *n*-alkanes** | | | |
| n-C21:0 | C27H56 | 380 | NIST |
| n-C23:0 | C29H60 | 408 | NIST |
| n-C25:0 | C31H64 | 436 | NIST |
| **Lignin oligomers** |  |  |  |
| Vinyl-guaiacol | C9H10O2 | 150 | NIST |
| Propenyl-guaiacol | C10H12O2 | 164 | NIST |
| Formyl-syringol | C9H10O4 | 182 | NIST |
| Allene-syringol | C11H12O3 | 192 | (15) |
| Propenyl-syringol | C11H14O3 | 194 | NIST |
| Methoxy-methylenzofuran | C10H10O2 | 162 | NIST |
|  |  |  |  |

# **Table S2. Pearson correlations of diatom, green algae, and vascular plant *rbc*L gene abundances in relation to lipid and lignin biomarkers and macromolecule contributions determined by pyrolysis-GC/MS.** (Note: *P<0.05; **P<0.01; ***P<0.001; ns: P>0.05).

|  | | | **Gene copies** | | | | | |
| --- | --- | --- | --- | --- | --- | --- | --- | --- |
|  | | | **Diatoms** | | **Green algae** | | **Vascular plants** | |
|  | | | **R^2^** | **P** | **R^2^** | **P** | **R^2^** | **P** |
| **Biomarkers** | Aquatic | Chlorophyll *a* | 0.72 | *** | 0.78 | *** | 0.00 | *ns* |
|  |  | Fatty acids C_14_+C_16_+C_18_ | 0.65 | *** | 0.86 | *** | 0.00 | *ns* |
|  |  | *n-*alkanes C_15_+C_17_+C_19_ | 0.50 | *** | 0.72 | *** | 0.00 | *ns* |
|  |  | Fatty acid C_20:5n-3_ | 0.71 | *** | 0.83 | *** | 0.00 | *ns* |
|  |  | Diatom sterols | 0.38 | *** | 0.58 | *** | 0.01 | *ns* |
|  |  | Fatty acid C_18:2n-6_ | 0.56 | *** | 0.82 | *** | 0.00 | *ns* |
|  | Terrestrial | *n-*alkanes C_27_+C_29_+C_31_ | 0.13 | *ns* | 0.26 | ** | 0.13 | *ns* |
|  |  | Fatty acids C_24_+C_26_+C_28_ | 0.07 | *ns* | 0.23 | ** | 0.04 | *ns* |
|  |  | Lignin | 0.01 | *ns* | 0.02 | *ns* | 0.10 | *ns* |
| **Macro-molecules** | Aquatic | Total chlorophyll | 0.38 | *** | 0.65 | *** | 0.01 | *ns* |
|  |  | Proteins | 0.54 | *** | 0.69 | *** | 0.00 | *ns* |
|  | Terrestrial | (poly)aromatics | 0.01 | *ns* | 0.02 | *ns* | 0.07 | *ns* |

# **Table S3. Differences in community compositions of diatoms, green algae and vascular plants in relation to (a) trophic state based on analysis of similarities (ANOSIM) among total communities, and (**b**) in relation to age based on Spearman correlations with relative abundances of taxonomic groups.** * P<0.05; ** P<0.01; *** P<0.001. False-positives were identified and removed using the Benjamini–Hochberg method. The deepest samples from Lake Greifen and Lake Zug were deposited when these lakes were still oligotrophic, and are counted as oligotrophic. Sediments of Lake Zurich from before 1980 are counted as eutrophic to match the trophic state at the time of deposition. *[Note to Table S3a: Mesotrophic samples were only recovered from Lake Zurich after 1980. Due to the small sample size of mesotrophic samples and absence of samples from lakes other than Lake Zurich, we focus on the eutrophic-oligotrophic comparison in the main text.]*

| **a** | **Diatoms** | | **Green algae** | | **Vascular plants** | | **18S rRNA gene** | |
| --- | --- | --- | --- | --- | --- | --- | --- | --- |
| **Comparisons** | **R** | **P** | **R** | **P** | **R** | **P** | **R** | **P** |
| Eutrophic-Mesotrophic* | 0.28 | * | 0.60 | ** | -0.06 | - | -0.14 | - |
| Eutrophic-Oligotrophic | 0.30 | *** | 0.03 | - | 0.17 | * | 0.15 | - |
| Mesotrophic*-Oligotrophic | 0.68 | *** | 0.47 | ** | -0.001 | - | -0.27 | - |

| **b**  **b** |  |  | **Sediment age** | |
| --- | --- | --- | --- | --- |
|  | **Taxonomic Groups** | | **R^2^** | **P** |
| **Diatoms (*rbc*L)** | *Stephanodiscaceae* | *Stephanodiscus* | 0.02 | - |
|  |  | *Discostella* | 0.12 | - |
|  |  | Unknown *Stephanodiscaceae* | 0.02 | - |
|  | *Fragilariales* | *Staurosira* | 0.01 | - |
|  |  | *Pseudostaurosira* | 0.21 | ** |
|  |  | *Fragilaria* | 0 | - |
|  | *Coscinodiscophyceae* | *Aulacoseira* | 0.33 | *** |
|  |  | Other *Coscinodiscophyceae* | 0.07 | - |
|  | *Naviculales* | *Fallacia* | 0.24 | ** |
|  |  | *Navicula* | 0.01 | - |
|  | Other *Asterionella* | | 0.02 | - |
|  | *Amphora* | | 0.11 | - |
|  | *Mallomonas* | | 0.32 | *** |
| **Green algae (*rbc*L)** | *Trebouxiophyceae* | *Choricystis* | 0.11 | * |
|  |  | *Choricystis* related cluster 2 | 0.26 | *** |
|  |  | *Choricystis* related cluster 1 | 0 | - |
|  | *Scenedesmaceae* | *Willea* | 0.05 | - |
|  |  | *Scenedesmus* *Desmodesmus* species cluster | 0.15 | * |
|  | *Chlorellales* | Unclassified *Chlorellaceae* Cluster 1 | 0.19 | ** |
|  |  | *Oocystis* | 0.01 | - |
| **Vascular plants**  **(*rbc*L)** | *Asteridae* | *Lamiales* | 0.02 | - |
|  |  | *Asterales* | 0.03 | - |
|  |  | *Apiales* | 0 | - |
|  |  | Unclassified *Asterid* | 0 | - |
|  | Dicot Angiosperms | *Fagales* | 0 | - |
|  |  | *Rosales* | 0.02 | - |
|  |  | *Sapindales* | 0 | - |
|  |  | *Caryophyllales* | 0.02 | - |
|  |  | *Proteales* | 0 | - |
|  |  | *Fabales* | 0 | - |
|  |  | *Saxifragales* | 0.01 | - |
|  |  | Unclassified Dicot Angiosperm | 0.05 | - |
|  | Monocot Angiosperms | *Poales* | 0 | - |
|  |  | *Asparagales* | 0.02 | - |
|  | Gymnosperms | *Pinales* | 0 | - |
| **All eukaryotes (18S rRNA genes)** | *Ochrophyta* | Diatoms | 0.31 | ** |
|  |  | *Chrysophyceae* | 0.24 | * |
|  |  | *Eustigmatophyceae* | 0.09 | - |
|  |  | *Dinoflagellata* | 0.01 | - |
|  |  | Green algae | 0.09 | - |
|  |  | Other*.Stramenopiles* | 0.22 | * |
|  | *Alveolata* | *Ciliophora* | 0.34 | ** |
|  |  | *Perkinsea* | 0.13 | - |
|  |  | *Other.Alveolata* | 0.31 | ** |
|  | *Opisthokonta* | *Fungi* | 0.40 | ** |
|  |  | *Metazoa* | 0.32 | ** |
|  | Vascular plants | | 0.64 | *** |
|  | *Cercozoa* | | 0.07 | - |
|  | *Conosa* | | 0.28 | * |

The onset of eutrophication was ~1920 in Lake Greifen, ~1890 in Lake Baldegg, ~1930 in Lake Zug, and ~1890 in Lake Zurich. The transition from eutrophic to mesotrophic in Zurich took place around 1980.

**Eutrophic**: all samples in Lake Greifen except for the bottom sample which was oligotrophic pre 1920, all samples in Lake Baldegg, all samples in Lake Zug except for the bottom sample which was oligotrophic pre 1930, and samples before 1980s in Lake Zurich.

**Mesotrophic**: samples in Lake Zurich after 1980.

**Oligotrophic**: all samples in Lake Lucerne, and the bottom samples in Lake Greifen and Lake Zug.

# **Table S4. Groups of diatoms, green algae and vascular plants, and 18S rRNA genes that are the most influential (70% of cumulative contribution) in driving community structure differences in relation to trophic states based on SIMPER analysis.** * P<0.05; ** P<0.01; *** P<0.001. Trophic classification is shown in Table S5. Average: Species contribution to average between-group dissimilarity. Cumsum: Ordered cumulative contribution. The deepest samples from Lake Greifen and Lake Zug were deposited when these lakes were still oligotrophic, and are counted as oligotrophic. Sediments of Lake Zurich from before 1980 are counted as eutrophic to match the trophic state at the time of deposition. *[Note: Mesotrophic samples were only recovered from Lake Zurich after 1980. Due to the small sample size of mesotrophic samples and absence of samples from lakes other than Lake Zurich, we focus on the eutrophic-oligotrophic comparison in the main text.]*

|  | **Groups** | **Eutrophic-Mesotrophic** | | | **Eutrophic-Oligotrophic** | | | **Mesotrophic-Oligotrophic** | | |
| --- | --- | --- | --- | --- | --- | --- | --- | --- | --- | --- |
|  |  | **Average (%)** | **Cumsum (%)** | **P** | **Average (%)** | **Cumsum (%)** | **P** | **Average (%)** | **Cumsum (%)** | **P** |
| **Diatoms** | *Stephanodiscus* | 25.77 | 32.73 | ***** | 25.15 | 32.95 | *** | 7.47 | 65.57 | - |
|  | *Staurosira* | 20.50 | 58.77 | - | 23.15 | 63.27 | *** | 17.09 | 29.38 | - |
|  | *Aulacoseira* | 12.84 | 75.08 | ****** | - | - | - | 13.58 | 52.73 | ******* |
|  | *Cyclotella* | - | - | - | 2.50 | 66.55 | - | - | - | - |
|  | *Asterionella* | - | - | - | 2.42 | 69.72 | - | - | - | - |
|  | *Amphora* | - | - | - | 0.20 | 72.34 | ** | 20.40 | 69.07 | - |
|  | *Pseudostaurosira* | - | - | - | - | - | - | 17.29 | 72.04 | ***** |
| **Green algae** | *Choricystis*-related cluster 2 | 18.45 | 46.67 | ***** | 9.27 | 85.88 | - | 16.69 | 96.81 | ***** |
|  | *Choricystis* | 17.48 | 90.91 | - | 10.77 | 46.14 | - | 16.90 | 48.71 | - |
| **Vascular plants** | *Lamiales* | 13.70 | 17.27 | - | 14.31 | 36.02 | - | 8.98 | 43.33 | - |
|  | *Fagales* | 14.44 | 311.70 | - | 17.31 | 19.72 | ** | 16.58 | 20.18 | - |
|  | *Sapindales* | 7.54 | 41.20 | - | 5.63 | 71.59 | - | - | - | - |
|  | *Poales* | 7.01 | 50.05 | - | - | - | - | 7.35 | 52.28 | - |
|  | *Asparagales* | 6.63 | 58.42 | - | - | - | - | 6.11 | 67.36 | - |
|  | Unclassif. *Asterids* | 6.60 | 66.74 | - | 8.88 | 58.73 | - | - | - | - |
|  | *Saxifragales* | 4.61 | 72.56 | ***** | - | - | - | - | - | - |
|  | *Rosales* | - | - | - | 11.05 | 48.61 | * | 10.03 | 32.40 | - |
|  | Unclassified Dicot Angiosperm | - | - | - | 5.66 | 65.17 | * | 5.76 | 74.38 | - |
| **18S rRNA genes** | *Fungi* | 14.31 | 27.50 | - | 11.80 | 18.92 | - | 11.32 | 21.25 | - |
|  | *Metazoa* | 7.95 | 42.78 | - | 11.72 | 37.71 | - | 6.89 | 35.80 | - |
|  | *Ciliophora* | 5.17 | 52.71 | - | 4.54 | 64.68 | - | - | - | - |
|  | Diatoms | 4.68 | 61.71 | - | 4.87 | 57.40 | - | 4.09 | 64.38 | - |
|  | Other *Stramenopiles* | 3.87 | 69.14 | - | - | - | - | - | - | - |
|  | Other *Alveolata* | 3.15 | 75.19 | - | 7.41 | 49.59 | * | 7.75 | 35.80 | - |
|  | Vascular plants | - | - | - | 4.27 | 71.52 | ** | 4.24 | 56.71 | - |
|  | Green algae | - | - | - | - | - | - | 3.53 | 71.01 | - |

# **Table S5. Descriptions of lakes included in this study.** All data are from Fiskal*, et al.* (16), which also provides detailed records of P concentration changes through time, and their likely drivers, in all five lakes. Sedimentation rates changed in Lake Zurich (25 m), from 0.23 cm yr^-1^ in the top 7 to 0.27 cm yr^-1^ below and in Lake Lucerne (45 m) from 0.16 cm yr^-1^ in the top 5 cm to 0.14 cm yr^-1^ below.

|  | **Area**  **(km^2^)** | **Water**  **residence time**  **(yr)** | **Station names**  **and water depths (m)** | **Sedimentation rate**  **(cm yr^-1^)** | **Trophic status** | **Bottom water [O_2_] (µM)** | **Onset of eutrophication (sediment depth (cm) and year)** | **Age of deepest sediment layer sampled (yr)** |
| --- | --- | --- | --- | --- | --- | --- | --- | --- |
| Lake Greifen | 8.4 | 0.148 | Shallow (15 m) | 0.29 | Eutrophic | seasonally hypoxic | 26-30, ~1920 | 1886 |
|  |  |  | Medium (24 m) | 0.31 |  |  | 28-32, ~1920 | 1893 |
|  |  |  | Deep (33 m) | 0.37 |  |  | 32-36, ~1920 | 1913 |
| Lake Baldegg | 5.2 | 0.173 | Shallow (21 m) | 0.29 | Eutrophic | 15.6-125 | > 38, ~1890 | 1884 |
|  |  |  | Medium (45 m) | 0.32 |  | 125-250 | > 38, ~1890 | 1922 |
|  |  |  | Deep (68 m) | 0.27 |  | 15.6–125 | > 38, ~1890 | 1877 |
| Lake Zug | 38 | 3.2 | Shallow (25 m) | 0.22 | Eutrophic | 125-250 | 18-20, ~1930 | 1840 |
|  |  |  | Medium (35 m) | 0.25 |  | 125-250 | 20-24, ~1930 | 1866 |
|  |  |  | Deep (50 m) | 0.37 |  | 125-250 | 28-32, ~1930 | 1912 |
| Lake Zurich | 67.3 | 3.9 | Shallow (25 m) | 0.22/0.27 | Mesotrophic | 15.6-125 | 32-36, ~1890 | 1875 |
|  |  |  | Medium (45 m) | 0.35 |  | 125-250 | 32-36, ~1890 | 1864 |
|  |  |  | Deep (137 m) | 0.20 |  | hypoxic | > 38, ~1890 | 1891 |
| Lake Lucerne | 116 | 11.9 | Shallow (24 m) | 0.17 | Oligotrophic | > 250 |  | 1839 |
|  |  |  | Medium (45 m) | 0.16/0.14 |  | > 250 |  | 1801 |
|  |  |  | Deep (93 m) | 0.06 |  | > 250 |  | 1403 |

# **Table S6. Depth intervals and corresponding ages of sediment samples analyzed in this study.** All sediment ages are from Fiskal*, et al.* (16)**.** Sample code example: 12-14/1971 = sediment interval from 12-14 cm, of which the center (13 cm) was deposited in 1971. Bold underlined samples were chosen for *rbc*L gene MiSeq sequencing. All sediment samples that date back to layers that were deposited prior to the approximate onset of eutrophication are marked with asterisks (*).

|  | **Lake Greifen** | | | **Lake Baldegg** | | | **Lake Zug** | | |
| --- | --- | --- | --- | --- | --- | --- | --- | --- | --- |
|  | **Shallow** | **Medium** | **Deep** | **Shallow** | **Medium** | **Deep** | **Shallow** | **Medium** | **Deep** |
| Depth(cm)/  Year | 0-0.5/2015 | 0-0.5/2015 | **0-0.5/2015** | 0-0.5/2015 | 0-0.5/2015 | **0-0.5/2015** | 0-0.5/2014 | 0-0.5/2015 | **0-0.5/2015** |
|  | 0.5-1/2013 | 0.5-1/2013 | 0.5-1/2013 | 0.5-1/2013 | 0.5-1/2013 | 0.5-1/2013 | 0.5-1/2012 | 0.5-1/2013 | 0.5-1/2013 |
|  | 1-1.5/2011 | 1-1.5/2011 | **1-1.5/2012** | 1-1.5/2011 | 1-1.5/2012 | 1-1.5/2011 | 1-1.5/2010 | 1-1.5/2011 | **1-1.5/2012** |
|  | 1.5-2/2010 | 1.5-2/2010 | 1.5-2/2011 | 1.5-2/2009 | 1.5-2/2010 | **1.5-2/2009** | 1.5-2/2007 | 1.5-2/2009 | 1.5-2/2011 |
|  | 2-3/2007 | 2-3/2007 | **2-3/2009** | 2-3/2007 | 2-3/2008 | 2-3/2006 | 2-3/2004 | 2-3/2006 | **2-3/2009** |
|  | 3-4/2004 | 3-4/2004 | 3-4/2006 | 3-4/2003 | 3-4/2005 | **3-4/2003** | 3-4/1999 | 3-4/2002 | 3-4/2006 |
|  | 4-6/1998 | 4-6/1999 | **4-6/2002** | 4-6/1998 | 4-6/2000 | **4-6/1997** | 4-6/1992 |  | **4-6/2002** |
|  | 6-8/1992 | 6-8/1993 | 6-8/1997 | 6-8/1991 | 6-8/1994 | 6-8/1990 | 6-8/1983 | 6-8/1988 | 6-8/1996 |
|  | 8-10/1985 | 8-10/1987 | **8-10/1991** | 8-10/1984 | 8-10/1987 | **8-10/1983** | 8-10/1974 | 8-10/1980 | **8-10/1991** |
|  | 10-12/1978 | 10-12/1980 | 10-12/1986 | 10-12/1978 | 10-12/1981 | **10-12/1975** | 10-12/1965 | 10-12/1972 | 10-12/1986 |
|  | 12-14/1971 | 12-14/1974 | **12-14/1980** | 12-14/1971 | 12-14/1975 | 12-14/1968 | 12-14/1956 | 12-14/1964 | **12-14/1980** |
|  | 14-16/1964 | 14-16/1967 | **14-16/1975** | 14-16/1964 | 14-16/1969 | **14-16/1961** | 14-16/1946 | 14-16/1957 | **14-16/1975** |
|  | 16-18/1957 | 16-18/1961 | 16-18/1970 | 16-18/1957 | 16-18/1962 | 16-18/1953 | 16-18/1937 | 16-18/1949 | 16-18/1969 |
|  | 18-20/1951 | 18-20/1954 | **18-20/1964** | 18-20/1950 | 18-20/1956 | **18-20/1946** | 18-20/1928* | 18-20/1941 | **18-20/1964** |
|  | 20-24/1940 | 20-24/1945 | **20-24/1956** | 20-24/1940 | 20-24/1947 | **20-24/1935** | 20-24/1914* | 20-24/1929* | **20-24/1956** |
|  | 24-28/1927 | 24-28/1932 | 24-28/1945 | 24-28/1926 | 24-28/1934 | 24-28/1920 | 24-28/1896* | 24-28/1913* | 24-28/1945 |
|  | 28-32/1913* | 28-32/1919* | **28-32/1934** | 28-32/1912 | 28-32/1922 | **28-32/1906** | 28-32/1877* | 28-32/1898* | **28-32/1934** |
|  | 32-36/1899* | 32-36/1906* | 32-36/1924 | 32-36/1898 |  | 32-36/1891 | 32-36/1859* | 32-36/1882* | 32-36/1923* |
|  | 36-40/1886* | 36-40/1893* | **36-40/1913*** | 36-40/1884* |  | **36-40/1877*** | 36-40/1840* | 36-40/1866* | **36-40/1912*** |

*Continued* ***Table S6.***

|  | **Lake Zurich** | | | **Lake Lucerne** | | |
| --- | --- | --- | --- | --- | --- | --- |
|  | **Shallow** | **Medium** | **Deep** | **Shallow** | **Medium** | **Deep** |
| Depth(cm)/  Year | 0-0.5/2014 | 0-0.5/2015 | **0-0.5/2014** | 0-0.5 | 0-0.5/2014 | **0-0.5/2011** |
|  | 0.5-1/2012 | 0.5-1/2014 | 0.5-1/2012 | 0.5-1/2013 | 0.5-1/2011 | 0.5-1/2003 |
|  | 1-1.5/2010 | 1-1.5/2011 | **1-1.5/2009** | 1-1.5/2008 | 1-1.5/2008 | **1-1.5/1995** |
|  | 1.5-2/2008 | 1.5-2/2009 | 1.5-2/2007 | 1.5-2/2005 | 1.5-2/2005 | 1.5-2/1986 |
|  | 2-3/2006 | 2-3/2006 | **2-3/2003** | 2-3/2001 | 2-3/1998 | 2-3/1975 |
|  | 3-4/2000 | 3-4/2002 | 3-4/1998 | 3-4/1995 | 3-4/1994 | **3-4/1959** |
|  | 4-6/1996 | 4-6/1996 | **4-6/1991** | 4-6/1986 | 4-6/1980 | **4-6/1935** |
|  | 6-8/1990 | 6-8/1988 | 6-8/1981 | 6-8/1974 | 6-8/1966 | 6-8/1903 |
|  | 8-10/1982 | 8-10/1980 | **8-10/1971** | 8-10/1963 | 8-10/1951 | **8-10/1870** |
|  | 10-12/1974 | 10-12/1972 | **10-12/1961** | 10-12/1951 | 10-12/1937 | **10-12/1838** |
|  | 12-14/1967 | 12-14/1964 | 12-14/1951 | 12-14/1939 | 12-14/1923 | 12-14/1807 |
|  | 14-16/1960 | 14-16/1956 | **14-16/1941** | 14-16/1927 | 14-16/1909 | **14-16/1774** |
|  | 16-18/1953 | 16-18/1948 | 16-18/1931 | 16-18/1916 | 16-18/1894 | 16-18/1742 |
|  | 18-20/1945 | 18-20/1940 | **18-20/1921** | 18-20/1904 | 18-20/1880 | 18-20/1710 |
|  | 20-24/1934 | 20-24/1928 | **20-24** | 20-24/1886 | 20-24/1858 | **20-24/1661** |
|  | 24-28/1919 | 24-28/1912 | 24-28 | 24-28/1862 | 24-28/1829 | 24-28 |
|  | 28-32/1904 | 28-32/1896 | **28-32** | 28-32/1839 | 28-32/1801 | **28-32** |
|  | 32-36/1890 | 32-36/1880* | 32-36 |  |  | 32-36 |
|  | 36-40/1875* | 36-40/1864* | **36-40/1891*** |  |  | **36-40/1403** |

**Table S7. Tested qPCR primers targeting *Ochrophyta* (diatoms) and *Chlorophyta* (green algae) *rbc*L genes. The Ochro-rbcL_43F / 432R *rbc*L primer pair, which we in the end used for diatom *rbc*L qPCR and sequencing, produced *rbc*L libraries consisting to ~98% of diatom *rbc*L sequences. The Chloro-rbcL_110F / 537R primer pair was used for green algae qPCR and sequencing.** *Ochrophyta* primers were designed for minimum degeneracy and target chloroplast *rbc*L genes of all *Ochrophyta* (with optimal coverage for diatoms and dinoflagellates). Since the vast majority of dinoflagellates in the lacustrine systems studied have highly divergent, nuclear-encoded “Proteobacteria-type” form II RuBisCo genes, the primer combination ended up being specific to diatoms. We chose the Ochro-rbcL_43F / 432R rbcL and Chloro-rbcL_110F / 537R primer combinations due to the presence of low concentrations of non-target PCR amplicon lengths with the other primer pairs. Furthermore, the primer pair Chloro-rbcL_537F / 1021R produces a 507 bp long amplicon that is difficult to sequence using the MiSeq paired-end 2 × 300 bp read technology because it exceeds the target length after addition of sequence adaptors and sample-specific indices. [Primer *in silico* coverages: Ochro-rbcL_43F: 0 mismatches with any of the 425 diatom sequences in database; Ochro-rbcL_432R: 0 to 1 mismatches with 99% of the diatom sequences in database; Chloro-rbcL_110F: 0 mismatches with 99% of the 370 green algal sequences in database; Chloro-rbcL_537R: 0-2 mismatches with 94% of the green algal sequences in the database.]

| **Targeted groups** | **Primers** | **Primer sequence (5´-3’)** | **Annealing temp. (°C)** | **Product length (bp)** | **Nucleotide position within gene (5’-3’)** | **Standards** |
| --- | --- | --- | --- | --- | --- | --- |
| *Ochrophyta* | Ochro-rbcL_43F | CGT TAC GAA TCT GGT GTA AT | 55 | 389 | 43-63 | *Stephanodiscus sp.* |
|  | Ochro-rbcL_432R | GGA ATA CGC ATA TCT TCT AAA CGT A |  |  | 412-432 |  |
|  | Ochro-rbcL_952F_mix | TGT AAA TGG ATG CGT ATG  TGT AAA TGG ATG AGA ATG | 58 | 225 | 952-976 | *Stephanodiscus sp.* |
|  | Ochro-rbcL_1177R | TTG GTG CAT TTG ACC ACA |  |  | 1159-1177 |  |
| *Chlorophyta* | Chloro-rbcL_110F | TWG CRG CWT TYC GTA TGA CIC | 54 | 427 | 110-131 | *Choricystis sp.* |
|  | Chloro-rbcL_537R | CCT AAK TTW GGT TTA ATN GTA CA |  |  | 514-537 |  |
|  | Chloro-rbcL_537F | TG TAC NAT TAA ACC WAA MTT AGG | 60 | 507 | 514-537 | *Choricystis sp.* |
|  | Chloro-rbcL_1021R | TTC WCG BTC ACC TTC TAR TTT |  |  | 1000-1021 |  |

# **Supplementary Figures**


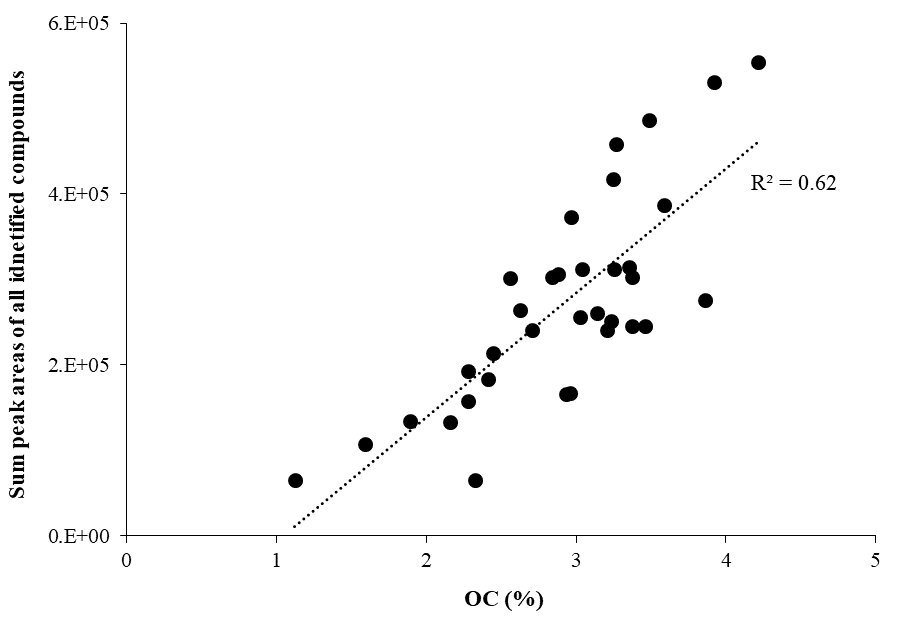


## **Fig. S1.** **Correlations between the sum of peak areas of all identified pyrolytic organic compounds and total OC content (p<0.01).** All OC content values are from Fiskal*, et al.* (16).


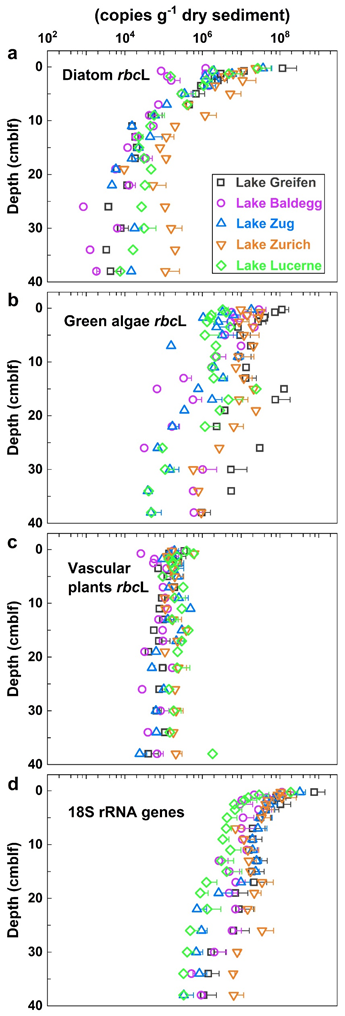


## **Fig. S2. Depth profiles of (a) diatom, (b) green algae, and (c) vascular plant *rbc*L copy numbers, and of (d) 18S rRNA gene copy numbers across the five lakes.** Each data point represents the average ± standard deviation (SD) of three stations. Lakes are listed in the legend in order of highest (eutrophic) to lowest (oligotrophic) present-day trophic state. This figure provides a general, first overview of depth trends across the cores sampled. Data in all later figures is plotted versus sediment age. Sediment age models of all sites were published previously (16) and are based on radionuclide (Pb-210 and Cs-137) measurements.

## **Fig. S3. Copy numbers of eukaryotic chloroplast *rbc*L of (a) diatoms, (b) green algae and (c) vascular plants, and (d) 18S rRNA genes across fifteen stations in five lakes ordered from most eutrophic (Lake Greifen) to oligotrophic (Lake Lucerne).**


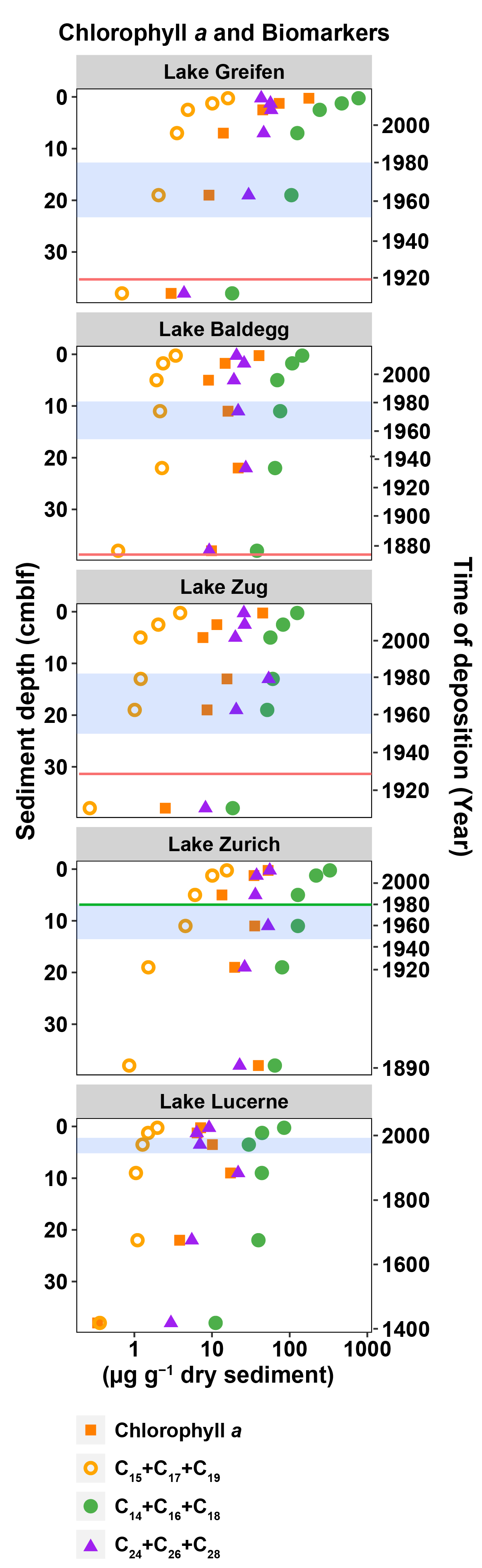


## **Fig. S4.** **Trends in contents of chlorophyll *a*, C_15_+C_17_+C_19_, C_14_+C_16_+C_18_, and C_24_+C_26_+C_28_ versus sediment depth and age.** Lakes are listed in order of trophic state, from most eutrophic (Lake Greifen) to oligotrophic (Lake Lucerne). The onset of eutrophication is indicated by the red lines (Lake Greifen: ~1920; Lake Baldegg: ~1890; Lake Zug: ~1930). The time period of peak eutrophication from 1950-1980 is indicated as a blue-shaded area. The transition from eutrophic to mesotrophic in Zurich took place around 1980 (green line) [Note: the increased sedimentation rates in Lake Zurich below 19 cm (~1920), which are the result of several large turbidites.]. Sediment age models of all sites based on extrapolated radionuclide (Pb-210_unsupported_ and Cs-137) measurements were published in Fiskal *et al.* (16).

## **Fig. S5. *rbc*L copies per gram of dry sediment versus sediment age in each lake (Power relationship).** a): Diatoms; b): Green algae; c): Vascular plants.

**b**

**a**

## **Fig. S6.** **18S gene copy numbers of diatoms, green algae, and vascular plants in relation to (a) sediment depth, and (b) sediment age.** All values calculated my multiplying sample-specific 18S rRNA gene read fractions with total 18S gene copy numbers.

## **Fig. S7.** **Trends in the decay rates of chlorophyll *a*, C_14_+C_16_+C_18_, C_15_+C_17_+C_19_, and C_24_+C_26_+C_28_ through time.**

***Continued Fig. S8***

## **Fig. S8**. **Linear relationships between *rbc*L copies of diatoms, green algae and vascular plants, and biomarkers and organic macromolecules.**


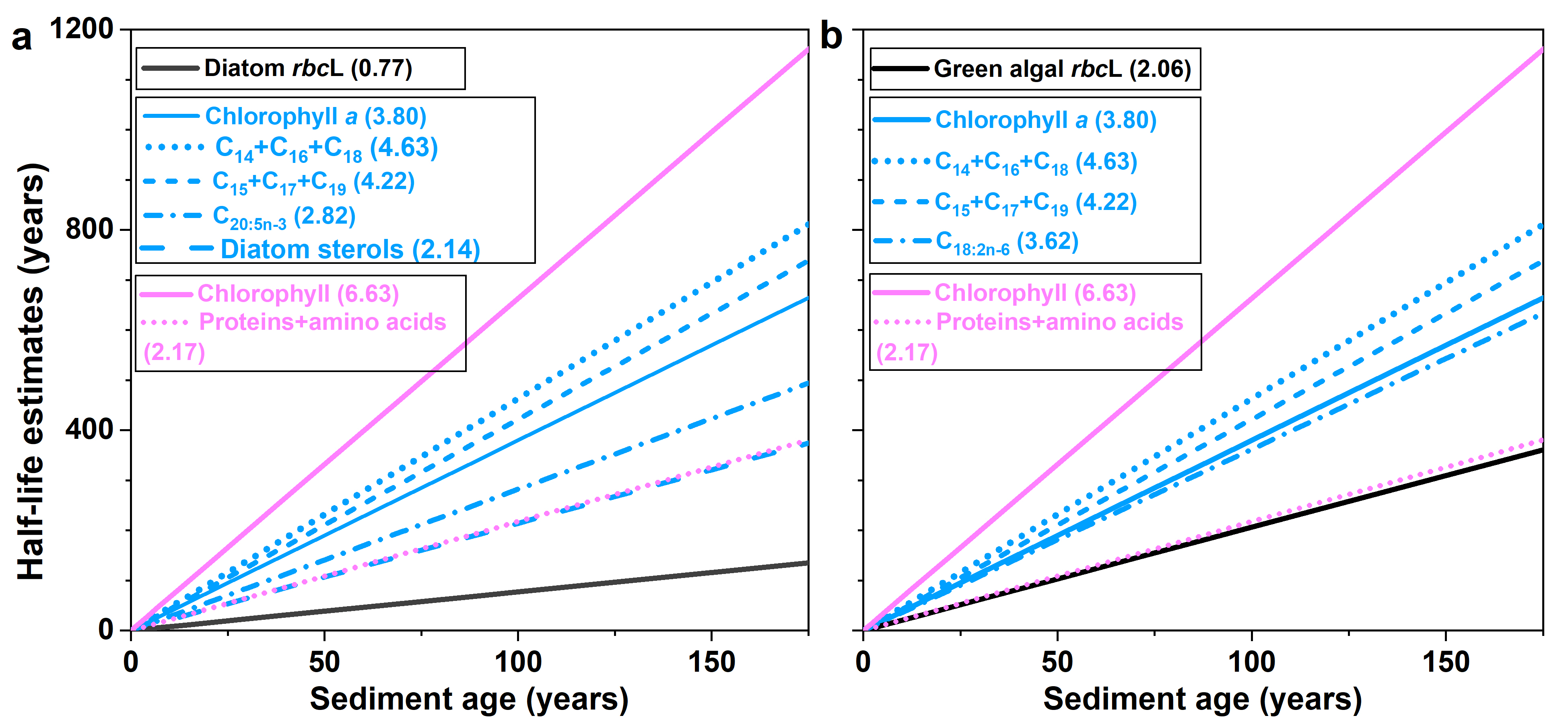


## **Fig. S9. Half-life estimates of (a) diatom and (b) green algal biomolecules in relation to sediment age.** Diatom sterols consist of brassicasterol + 24-methylenecholesterol.


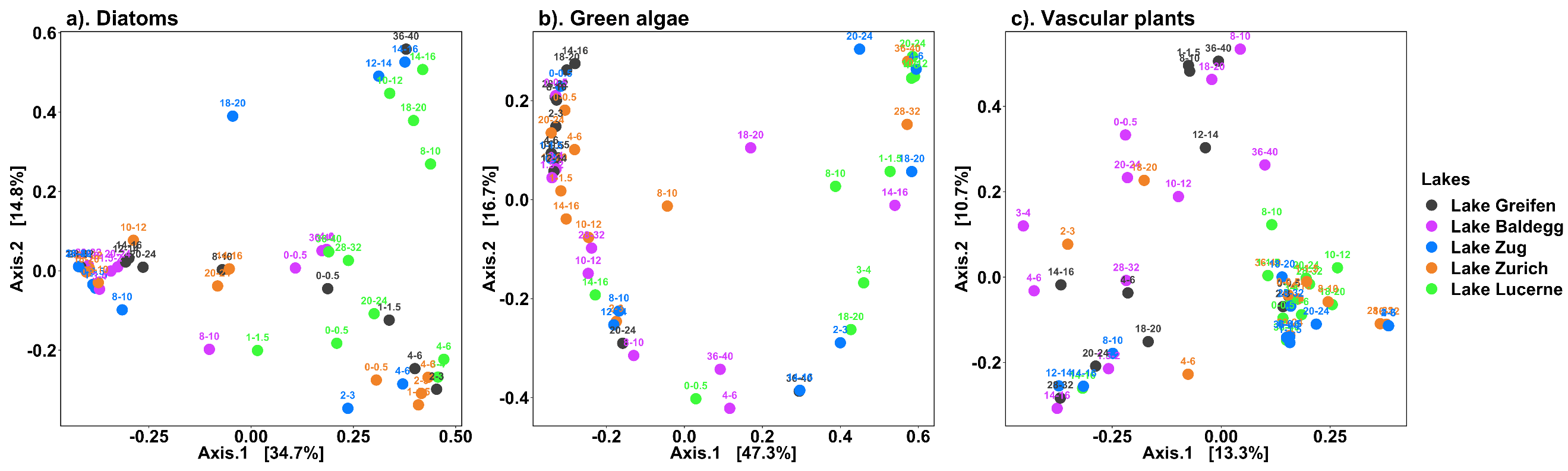


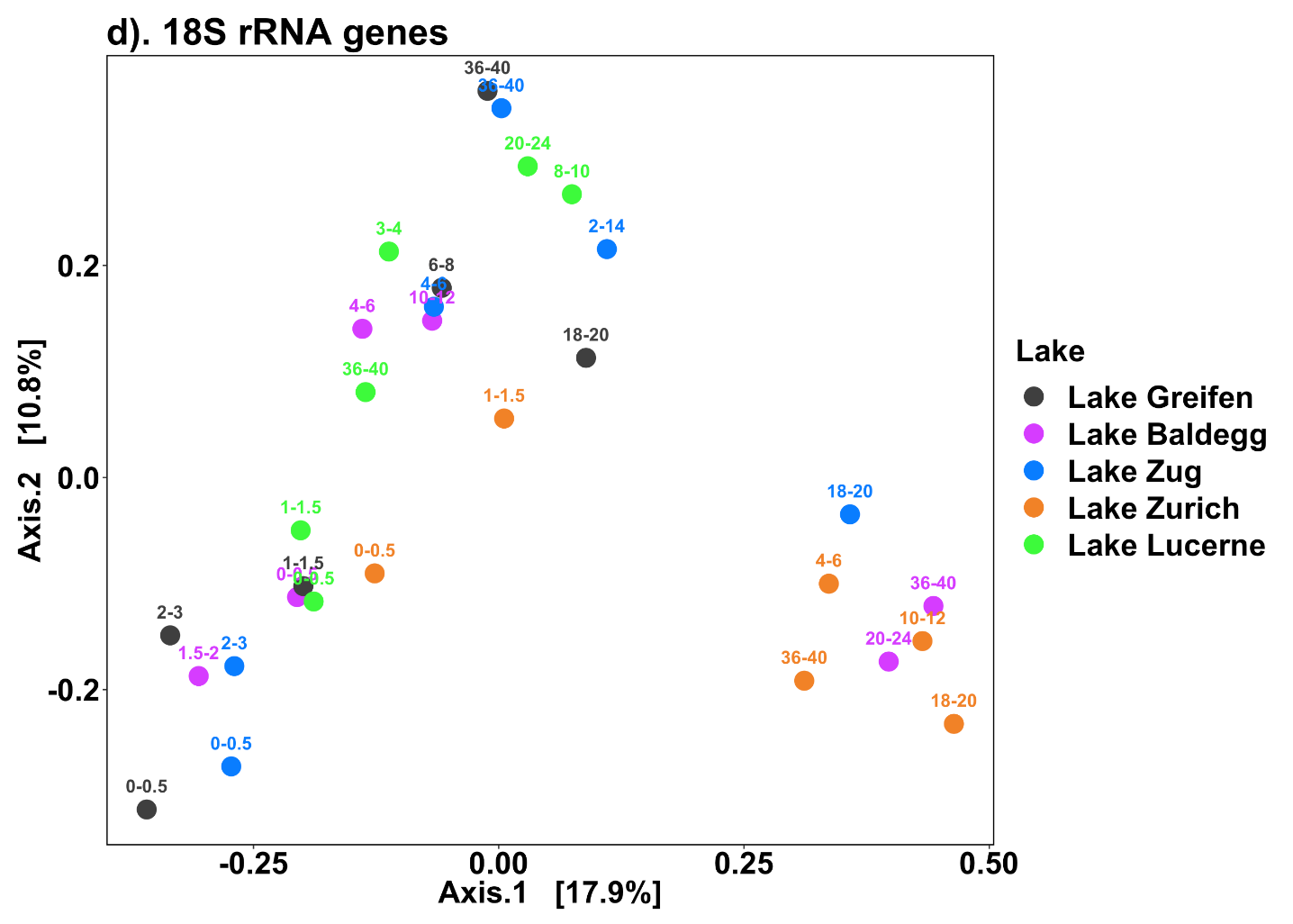


**Fig. S1**0. **PCoA (Principal Coordinates Analysis) of diatoms (a), green algae (b) and (c) vascular plants, (d) 18S rRNA genes on a 97% ZOTU level based on Bray-Curtis dissimilarity.** The numbers next to the shapes indicate the sediment depths, for example, 0–0.5 denotes 0–0.5 cm sediment depth.


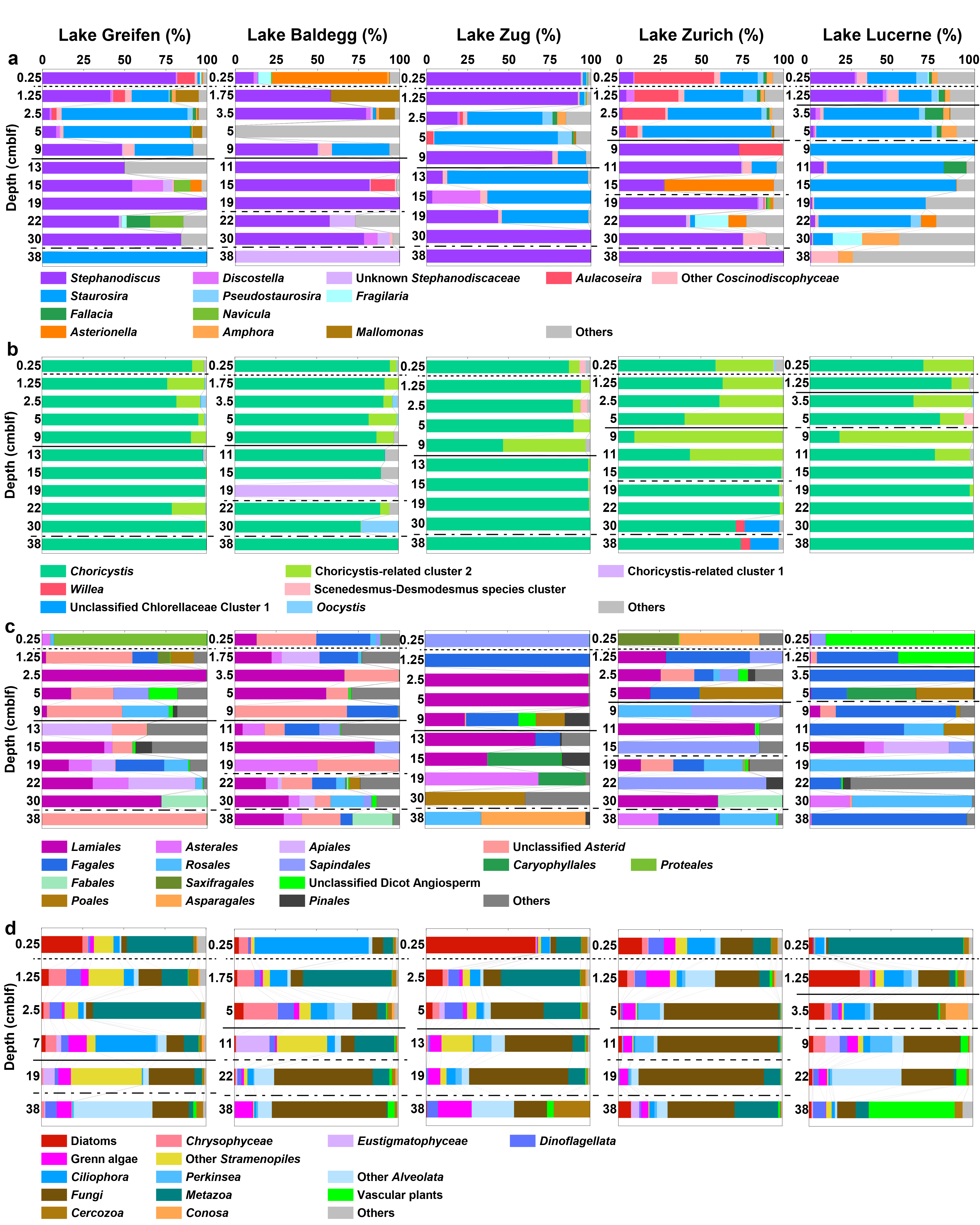


## **Fig. S11.** **Depth profiles of relative abundances of dominant groups of (a) diatoms, (b) green algae, and (c) vascular plant *rbc*L sequences, as well as (d) 18R rRNA gene sequences.** All data are from the deepest sampling stations in each lake. Graphs are ordered from most eutrophic (Lake Greifen) to oligotrophic (Lake Lucerne). The bottom dot-dashed line indicates the samples below were from pre 1900, among which bottom sediments from Lake Greifen and Lake Zug were from pre-eutrophic time. Samples between dot-dashed and dashed lines were from pre 1940. Samples between dashed and black lines were between 1940 and 1980s. Samples between black and dotted lines were after 1980s.

**a**

**b**

| **Pearson correlation** | Sediment depth | | | Sediment age | | |
| --- | --- | --- | --- | --- | --- | --- |
|  | R | R^2^ | P | R | R^2^ | P |
| C/V | -0.57 | 0.33 | <0.001 | -0.49 | 0.25 | <0.01 |
| S/V | -0.39 | 0.15 | <0.05 | -0.40 | <0.20 | <0.01 |

## **Fig. S12. (a) Relationships between the lignin parameters S/V and C/V, and their general plant sources. (b) Statistical relationships between C/V and S/V vs. sediment depth and sediment age based on Pearson correlations.** S/V is the sum of syringyl phenols (S) divided by the sum of vanillyl phenols (V), and reflects the relative abundances of lignin from gymnosperms, such as ferns and conifers (S/V = 0), and angiosperms, such as herbs, grasses, and hardwoods (S/V> 0). C/V is the sum of *p*-coumaric acid and ferulic acid (C) divided by the sum of vanillyl phenols (V). C/V ratios reflect the relative contributions of woody (C/V = 0) and non-woody (C/V>0) plant tissues (leaves, needles, barks, and stems)^13^. Numbers in the figure represent sediment depths (cm).


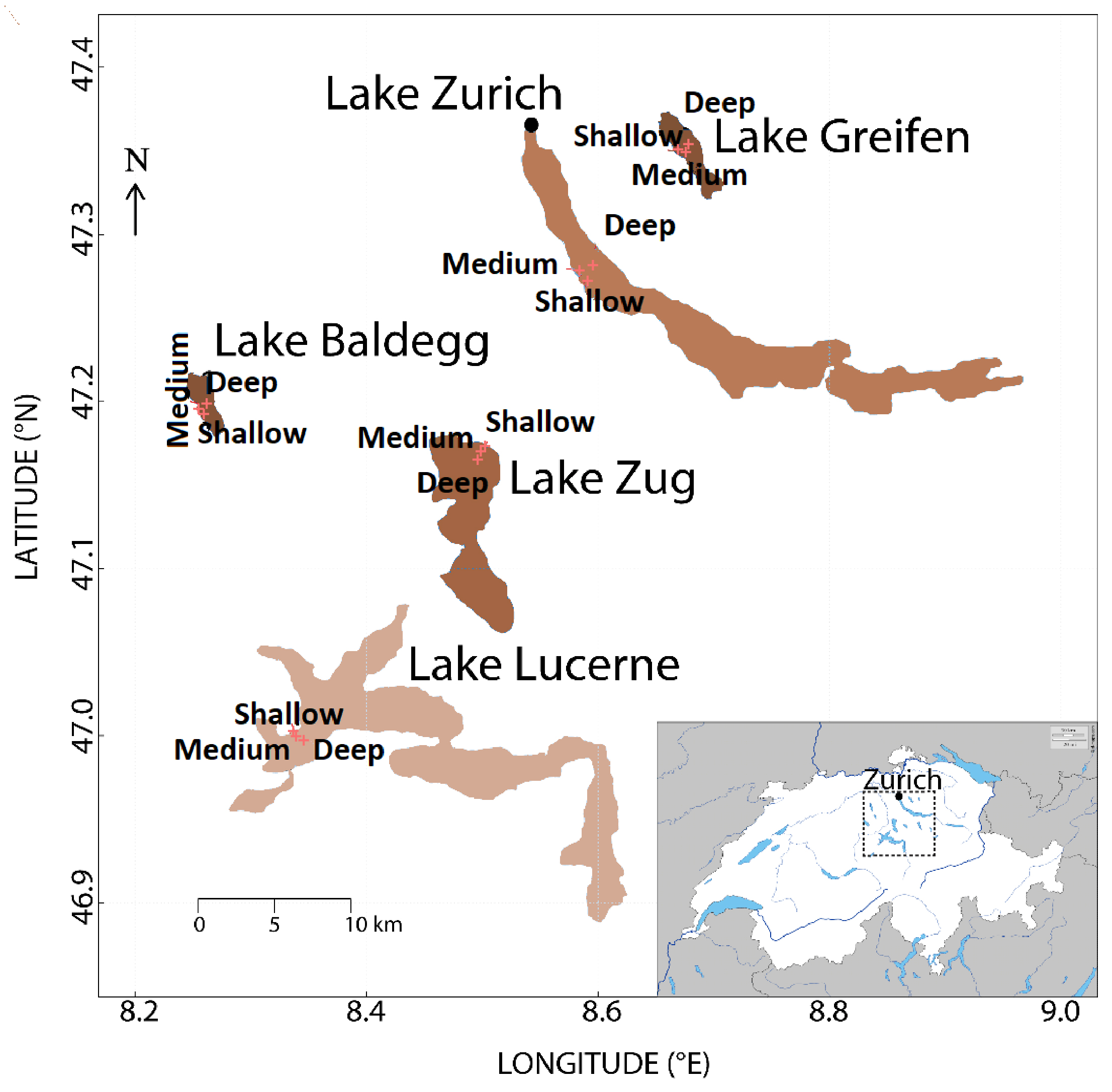


## **Fig. S13.** **Map of the sampling region in central Switzerland and the five lakes that were studied here** (16, 17). (Swiss map is adapted from <https://www.d-maps.com/carte.php?num_car=2648&lang=en>**).** Color indicates trophic state from light brown (oligotrophic) to brown (eutrophic).

# **References**

1. S. Naeher *et al.*, Impact of recent lake eutrophication on microbial community changes as revealed by high resolution lipid biomarkers in Rotsee (Switzerland). *Org Geochem* **49**, 86-95 (2012).

2. P. A. Cranwell, Lipids of Aquatic Sediments and Sedimenting Particulates. *Prog Lipid Res* **21**, 271-308 (1983).

3. P. A. Cranwell, G. Eglinton, N. Robinson, Lipids of Aquatic Organisms as Potential Contributors to Lacustrine Sediments .2. *Org Geochem* **11**, 513-527 (1987).

4. P. A. Meyers, R. Ishiwatari, Lacustrine Organic Geochemistry - an Overview of Indicators of Organic-Matter Sources and Diagenesis in Lake-Sediments. *Org Geochem* **20**, 867-900 (1993).

5. J. K. Volkman, Sterols in microorganisms. *Appl Microbiol Biot* **60**, 495-506 (2003).

6. S. W. Rampen, B. A. Abbas, S. Schouten, J. S. S. Damste, A comprehensive study of sterols in marine diatoms (Bacillariophyta): Implications for their use as tracers for diatom productivity. *Limnol Oceanogr* **55**, 91-105 (2010).

7. J. K. Volkman *et al.*, Microalgal biomarkers: A review of recent research developments. *Org Geochem* **29**, 1163-1179 (1998).

8. S. M. Budge, C. C. Parrish, Lipid biogeochemistry of plankton, settling matter and sediments in Trinity Bay, Newfoundland. II. Fatty acids. *Org Geochem* **29**, 1547-1559 (1998).

9. M. A. Goñi, S. Montgomery, Alkaline CuO oxidation with a microwave digestion system: Lignin analyses of geochemical samples. *Anal Chem* **72**, 3116-3121 (2000).

10. J. Tolu, L. Gerber, J. F. Boily, R. Bindler, High-throughput characterization of sediment organic matter by pyrolysis-gas chromatography/mass spectrometry and multivariate curve resolution: A promising analytical tool in (paleo) limnology. *Anal Chim Acta* **880**, 93-102 (2015).

11. L. Gerber, M. Eliasson, J. Trygg, T. Moritz, B. Sundberg, Multivariate curve resolution provides a high-throughput data processing pipeline for pyrolysis-gas chromatography/mass spectrometry. *J Anal Appl Pyrol* **95**, 95-100 (2012).

12. S. Ninnes, J. Tolu, C. Meyer-Jacob, T. M. Mighall, R. Bindler, Investigating molecular changes in organic matter composition in two Holocene lake-sediment records from central Sweden using pyrolysis-GC/MS. *J Geophys Res-Biogeo* **122**, 1423-1438 (2017).

13. D. Fabbri, A. Adamiano, G. Falini, R. De Marco, I. Mancini, Analytical pyrolysis of dipeptides containing proline and amino acids with polar side chains. Novel 2,5-diketopiperazine markers in the pyrolysates of proteins. *J Anal Appl Pyrol* **95**, 145-155 (2012).

14. R. T. Nguyen *et al.*, Preservation of algaenan and proteinaceous material during the oxic decay of Botryococcus braunii as revealed by pyrolysis-gas chromatography/mass spectrometry and C-13 NMR spectroscopy. *Org Geochem* **34**, 483-497 (2003).

15. O. Faix, D. Meier, I. Fortmann, *In Thermal degradation products of wood: gas chromatographic separation and mass spectrometric characterization of monomeric lignin derived products* (Springer, 1990), vol. 281-285.

16. A. Fiskal *et al.*, Effects of eutrophication on sedimentary organic carbon cycling in five temperate lakes. *Biogeosciences* **16**, 3725-3746 (2019).

17. X. G. Han, C. J. Schubert, A. Fiskal, N. Dubois, M. A. Lever, Eutrophication as a driver of microbial community structure in lake sediments. *Environ Microbiol* **22**, 3446-3462 (2020).
